# Free Energy Landscapes and Metastability in Methane Adsorption within a Representative Metal–Organic Framework

**DOI:** 10.1021/acsomega.5c09062

**Published:** 2025-12-17

**Authors:** Anthony Dorhauer, Malgorzata Stankiewicz, Bartosz Mazur, Bogdan Kuchta, Carlos Wexler

**Affiliations:** † Deptartment of Physics & Astronomy, 14716 University of Missouri, Columbia, Missouri 65211, United States; ‡ Materials Science & Engineering Institute, University of Missouri, Columbia, Missouri 65211, United States; § Faculty of Chemistry, 214839 Wroclaw University of Science and Technology, Wybrzeze Wyspianskiego 27, 50-370 Wroclaw, Poland; ∥ Department of Micro, Nano, and Bioprocess Engineering, Wroclaw University of Science and Technology, 50-370 Wrocław, Poland; ⊥ MADIREL, Aix-Marseille University, 13013 Marseille, France

## Abstract

Metal–organic frameworks (MOFs) are promising crystalline materials for gas storage due to their tunable porosity and high surface area. Adsorption in these materials exhibits complex behavior arising from confinement. We investigate methane (CH_4_) adsorption in an IRMOF-8 model under subcritical conditions using Grand Canonical Monte Carlo (GCMC) and Transition-Matrix Monte Carlo (TMMC) simulations. The uptake *N* displays sharp transitions between low- and high-density adsorption states, reflecting underlying metastable configurations separated by free-energy barriers. In GCMC, slow fluctuations and hysteresis complicate equilibrium characterization, while TMMC—using ghost particle insertions/deletions in the canonical ensemble—enables direct computation of the free-energy profile Ω­(*N*), revealing both stable and metastable adsorption states. These metastable states give rise to the hysteresis observed in GCMC. We also show that the uptake transitions correspond to cooperative rearrangements in the adsorbed phase, driven by competing adsorbate–adsorbate and adsorbate–framework interactions. To our knowledge, this is the first quantitative TMMC mapping of Ω­(*N*) for methane in IRMOF-8 that explicitly links free-energy minima to a three-state structural rearrangement of the confined CH_4_ phase. Although the simulations employ a rigid model framework the observed free-energy landscapes and cooperative rearrangements of the adsorbed phase illustrate general features of methane adsorption in nanoporous solids. These results clarify the thermodynamic origin of metastability in confined adsorption and provide a transferable framework for analyzing complex adsorption phenomena in porous materials.

## Graphical abstract

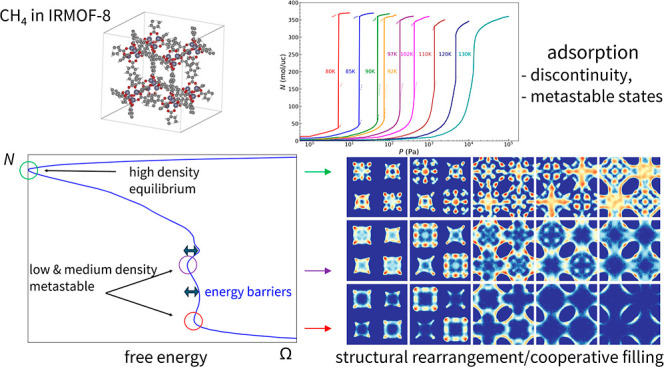

## 1 Introduction

Metal–organic frameworks (MOFs) are crystalline nanoporous materials with diverse applications, including gas storage (e.g., hydrogen, methane, and carbon dioxide), drug delivery, catalysis, and environmental remediation.^1–3^ Their high surface area, tunable chemistry, and structural diversity make them especially promising for gas capture and separation. Fluids confined within MOFs often exhibit thermodynamic behavior that deviates markedly from bulk phases. For example, phase coexistence points (e.g., vapor–liquid transitions) can shift significantly under nanoconfinement.^4–6^

Experimental and computational studies of CO_2_ and CH_4_ adsorption in IRMOF-1 and related frameworks have reported sharp uptake transitions in subcritical conditions, where the number of adsorbed molecules *N* increases steeply over a narrow pressure window; these transitions are attributed to collective structural rearrangements of the adsorbate phase.^7–14^

Grand Canonical Monte Carlo (GCMC) simulations are commonly used to model such adsorption phenomena^5^ but become problematic when the free energy landscape Ω­(*N*) exhibits multiple local minima separated by large barriers. Since the probability of crossing a barrier of height *W*_b_ scales as $e^{-\beta W_{b}}$ , where β = 1/*k*_B_ *T*, the system can become trapped in metastable states, leading to unrealistic hysteresis between adsorption and desorption branches and poor sampling.^15,16^

To overcome these limitations, we employ the Transition-Matrix Monte Carlo (TMMC) method,^17,18^ which indirectly samples the *grand canonical ensemble* by performing *canonical* simulations at fixed *N*, augmented by “ghost swaps”—particle insertion/removal attempts (*N* → *N* ± 1) that are tracked but ultimately not accepted. These attempted moves enable precise computation of the full free energy profile Ω­(*N*), allowing identification of equilibrium and metastable states, as well as energy barriers that govern adsorption kinetics and hysteresis.^12^ We follow similar investigations performed for adsorption in MOFs—including methodological discussion and sampling limitations.^19–21^

In this study we combine GCMC and TMMC to study CH_4_ adsorption in IRMOF-8. Figure 1 shows the IRMOF-8 unit cell (constructed from longer biphenyl linkers than IRMOF-1, with larger “cages” and “windows”). The larger topology naturally supports multistate adsorption and cooperative filling pathways and can amplify cooperative adsorption effects relative to IRMOF-1.^22,23^ We analyze adsorption isotherms, free energy profiles, and spatial density maps of CH_4_, revealing a third metastable state and its structural origin. At higher temperatures, we observe merging of local minima, smoothing of the uptake transition, and disappearance of associated structural rearrangements. To the best of our knowledge, this is the first study to quantify the complete free energy landscape of subcritical methane adsorption in IRMOF-8, other studies involving IRMOF-8 were either supercritical and/or involved different adsorbates.^24,25^ Furthermore, here we report how these result in a three-state cooperative rearrangement of the confined CH_4_ phase in a MOF.

**Figure 1:**
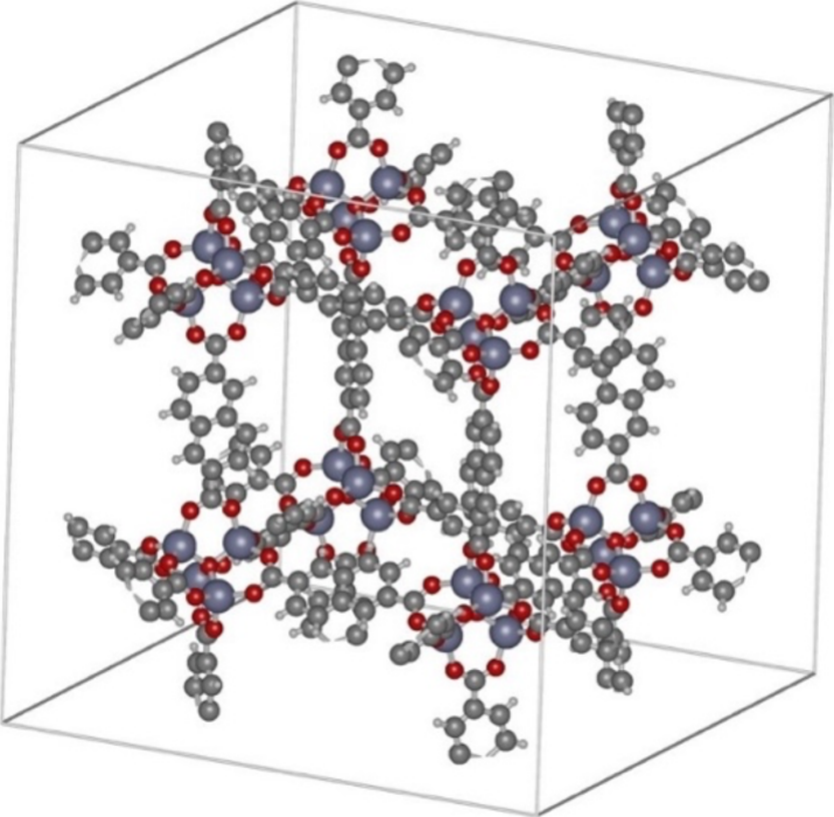
Unit cell of IRMOF-8. Atom colors: Zn (purple), O (red), C (dark gray), H (light gray).

The remainder of the paper is organized as follows: Section 2 gives simulation details, Section 3 presents results and discussion (isotherms, Ω­(*N*), structural maps, and temperature dependence), and Section 4 summarizes our conclusions and outlook.

## 2 Computational Methods and Simulation Parameters

We investigated methane (CH_4_) adsorption in a single unit cell of IRMOF-8 (Figure 1), which contains large pores interconnected by smaller windows. The IRMOF-8 structural model was taken from experimental crystallographic data.^7–9^ This topology creates distinct adsorption environments that favor cooperative effects at subcritical conditions. The framework consists of eight Zn_4_O nodes connected by naphthalene-2,6-dicarboxylate linkers,^26^ forming a cubic cell with dimensions 30.0915 Å × 30.0915 Å × 30.0915 Å. The framework was treated as rigid throughout all simulations; previous molecular dynamics and MC studies on the IRMOF family (e.g., Mason et al.,^27^ Coudert^19^) show that framework flexibility can alter quantitative barrier heights and transition pressures but typically does not eliminate cooperative adsorption physics in MOFs with large, interconnected pores.

Interactions were modeled using the 6–12 Lennard-Jones (LJ) potential

$$U\left(r\right)=\varepsilon_{ij}\left[{\left(\frac{\sigma_{ij}}{r_{ij}}\right)}^{12}-{\left(\frac{\sigma_{ij}}{r_{ij}}\right)}^{6}\right]$$ 1

with atom-specific parameters listed in Table 1. For the IRMOF-8 the universal force field (UFF)^28^ was used for Zn, and Dreiding^29^ for O, C, and H. Methane was modeled as a single-site pseudoatom with a molecular mass of 16.04246 amu. Because of its neutrality and symmetry, CH_4_ molecules have negligible electrostatic interactions (due to the lack of monopole, dipole or quadrupole electric moments), thus only LJ interactions were considered; parametrized by the diffuse-accessible-center nonbonded interaction sites (DACNIS) model.^30^ Cross-interactions were computed using Lorentz–Berthelot mixing rules $\left[\varepsilon_{ij}=\sqrt{\varepsilon_{i}\varepsilon_{j}},\;\sigma_{ij}=\left(\sigma_{i}+\sigma_{j}\right)/2\right]$ .^31,32^

**Table 1:** Lennard-Jones Parameters Used in Simulations

atom	ε (K)	σ (Å)
Zn	62.40	2.46
O	48.16	3.03
C	47.86	3.47
H	7.65	2.85
CH_4_	158.50	3.65

All simulations were performed using the RASPA2 molecular simulation package,^33^ applying periodic boundary conditions in all three directions. A spherical cutoff of 14 Å was used for the LJ interactions. Simulations were conducted at temperatures ranging from 80 to 130 K (80, 85, 90, 92, 97, 102, 110, 120, and 130 K), and pressures from 0 to 100 kPa. The CH_4_ fugacity was computed from the pressure using its critical properties: *T*_c_ = 190.564 K, *P*_c_ = 4599.2 kPa, and acentric factor ω = 0.01142 using the Peng-Robinson equation of state.^34^ GCMC and TMMC simulations were run for 2,000,000 cycles, with the first 200,000 used for initialization. GCMC simulations were used to observe particle number fluctuations and potential hysteresis. Adsorption and desorption branches were explored by initializing simulations with low (*N* ≈ 0) and high (*N* ≈ 370) loadings, respectively.

To compute the macrostate-dependent free energy profile Ω­(*N*), we employed the Transition-Matrix Monte Carlo (TMMC) method,^17,18^ implemented in a modified version of RASPA.^12,35^ In TMMC, simulations are carried out at fixed *N* (canonical ensemble) while introducing “ghost swap” moves, which attempt to change the particle number *N* → *N* ± 1. Although these moves are never accepted, their acceptance probabilities are calculated using standard GCMC Metropolis criteria

2 $$\begin{array}{l} p\left(N\to N+1\right)=\frac{V\Lambda^{-3}}{N+1}e^{\beta \mu }e^{-\beta \left(U_{N+1}-U_{N}\right)},\\ p\left(N\to N-1\right)=\frac{N\Lambda^{3}}{V}e^{-\beta \mu }e^{-\beta \left(U_{N-1}-U_{N}\right)}, \end{array}$$

where the chemical potential is determined by the pressure (fugacity *f*) by μ ∝ *k*_B_ *T* ln *f*. These attempt probabilities are accumulated in a transition matrix, which is normalized to estimate transition probabilities *P*(*N* → *N*, *N* ± 1). Using detailed balance^17,18^

3 Π(N)P(N→N+1)=Π(N+1)P(N+1→N)

the change of *free energy* for varying *N* can be computed by

4 $${\Delta \Omega_{N,N^{\prime }}\equiv \Omega \left(N^{\prime }\right)-\Omega \left(N\right)=k}_{B}T\ln\left[\Pi \left(N^{\prime }\right)/\Pi \left(N\right)\right]$$

By integration, the full free energy profile Ω­(*N*) at a given temperature and pressure is obtained. Furthermore, using macrostate reweighting, free energy landscapes for a range of fugacities can be constructed from a single TMMC simulation

5 $$\ln\Pi \left(N;\mu ,V,T\right)=\ln\Pi \left(N;\mu_{0},V,T\right)+\beta \left(\mu -\mu_{0}\right)+C$$

where, Π­(*N*;μ,*V*,*T*) is the macrostate probability distribution, β­(μ–μ_0_) is the reweighting term, and *C* is a constant independent of *N*.

## 3 Results and Discussion

Figure 2a,c present free energy profiles Ω­(*N*) for CH_4_ adsorption in IRMOF-8 at *T* = 80 K, computed using TMMC simulations for pressures ranging from 4 to 6.75 Pa (eqs 4 and 5). These profiles reveal three distinct regimes: (i) at low pressures (*P* ≲ 5 Pa), the global minimum occurs at *N* ∼ 0–40 molecules/unit cell, with a secondary minimum at *N* ∼ 370; (ii) at higher pressures (*P* ≳ 5.5 Pa), the global minimum shifts to *N* ∼ 370, and *two* metastable local minima appear at lower and intermediate occupancies (*N* ∼ 40 and *N* ∼ 160); and (iii) near the transition region, the barriers *W*_b_ between these minima diminish, indicating enhanced likelihood of phase transitions.

**Figure 2:**
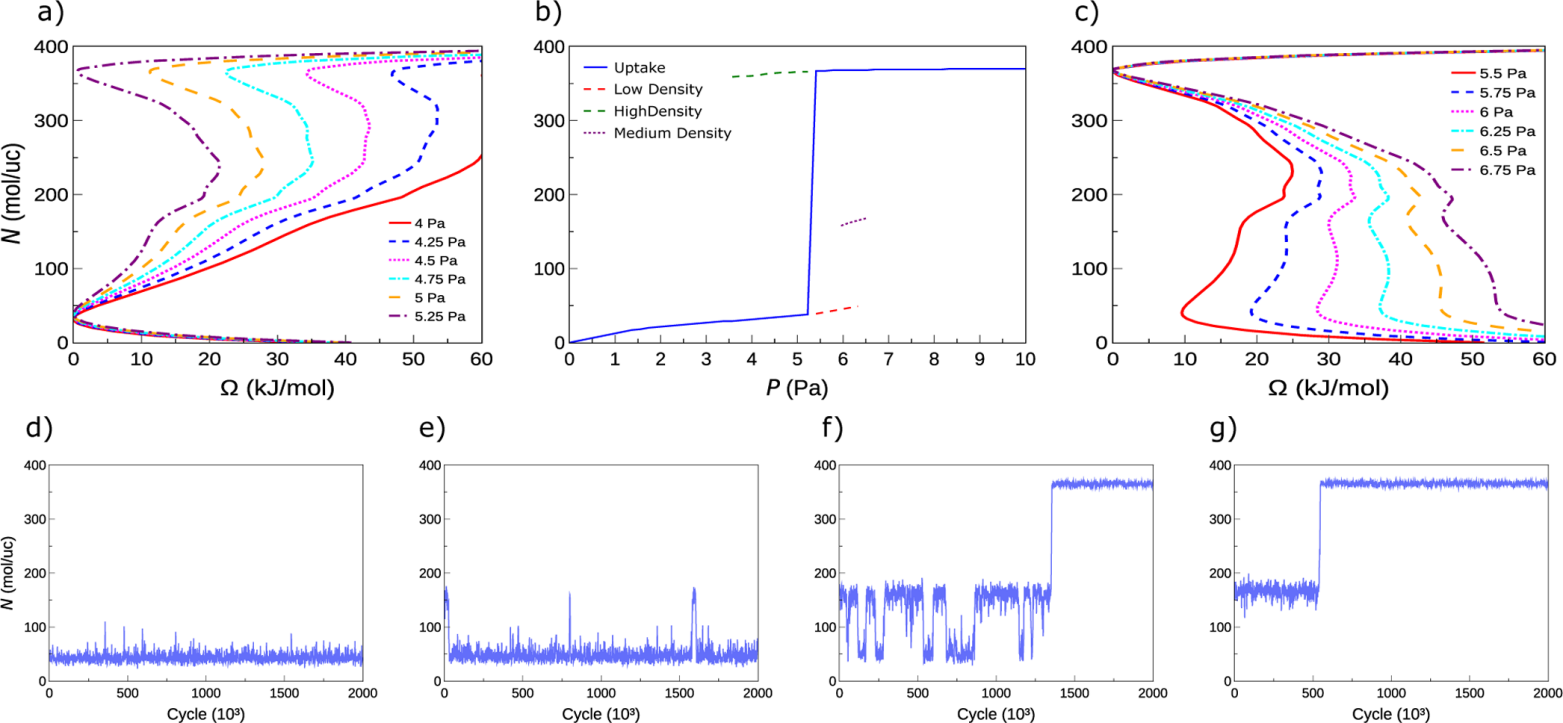
Free energy profiles Ω­(*N*) at low (a) and high (c) pressures for *T* = 80 K. (b) Corresponding CH_4_ isotherm with equilibrium occupancy and metastable states marked. GCMC fluctuations in *N* are shown in panels at *P* = 5.75 Pa (d), 6.0 Pa (e), 6.25 Pa (f), and 6.5 Pa (g). Energy profiles and adsorption isotherms for other temperatures may be found in the Supporting Information.

The corresponding adsorption isotherm at 80 K (Figure 2b), corresponding to the location of the minimum of Ω­(*N*) at each pressure, shows a sharp uptake transition near *P* ≃ 5.25 Pa, with the equilibrium occupancy shifting sharply from *N* ∼ 35 to ∼ 365. Additional local minima identified from TMMC are also shown and represent metastable states. These states are inaccessible experimentally because of their long time scales but are relevant for simulations using GCMC, as they may dominate the sampling and hinder adequate sampling.^12,13,16–18^

This metastability is evident in GCMC “adsorption branch” simulations initialized from low loadings. As shown in Figure 2d–g, the system remains trapped near *N* ∼ 40 even at *P* = 5.75 Pa, even though the thermodynamic state at this high-pressure regime should be at *N* ≃ 365. At *P* = 6.0 Pa, brief excursions to *N* ≈ 160 are observed, and by *P* = 6.25 Pa, the system samples the mid-density state frequently before transitioning to the thermodynamically favorable high density after ∼1.3 million cycles. At *P* = 6.5 Pa, the system reaches the mid-density state rapidly and then transitions fully to high density by 500,000 cycles.

The transition frequency (Figure 2d–g) is dictated by the barrier heights *W*_b_. At *P* < 6 Pa, *W*_b_ greatly exceeds *k*_B_ *T* (0.665 kJ/mol at 80 K), suppressing transitions. At *P* = 6.25 Pa, the barrier between the low- and mid-density states is a few *k*_B_ *T*, enabling transitions and coexistence. Once the high-density state is reached, however, the reverse barrier becomes large (∼16 *k*_B_ *T*), effectively preventing back-transitions. Thus, Markov chain averaged GCMC results may be misleading without enhanced sampling techniques such as TMMC.

Similar observations can be obtained in the “desorption swing” of a GCMC simulation (i.e., starting from *N* ∼ 370): a persistent high-density regime is seen to remain until very low pressures (though no intermediate density phase is observed in this regime), see Supporting Information.

Figure 3 shows CH_4_ adsorption isotherms from TMMC simulations over *T* = 80–130 K. At low temperatures (*T* ≲ 92 K), three distinct free energy minima are found near the transition pressure. As temperature increases, the low- and mid-density minima merge. By *T* ∼ 120–130 K, the remaining minima coalesce, and the uptake becomes continuous. This resembles critical phenomena, where distinct phase boundaries vanish as distinct free energy minima merge. Notably, these critical-like points occur well below the bulk critical temperature of bulk methane (*T*_c_ = 190.564 K), indicating strong confinement effects.

**Figure 3:**
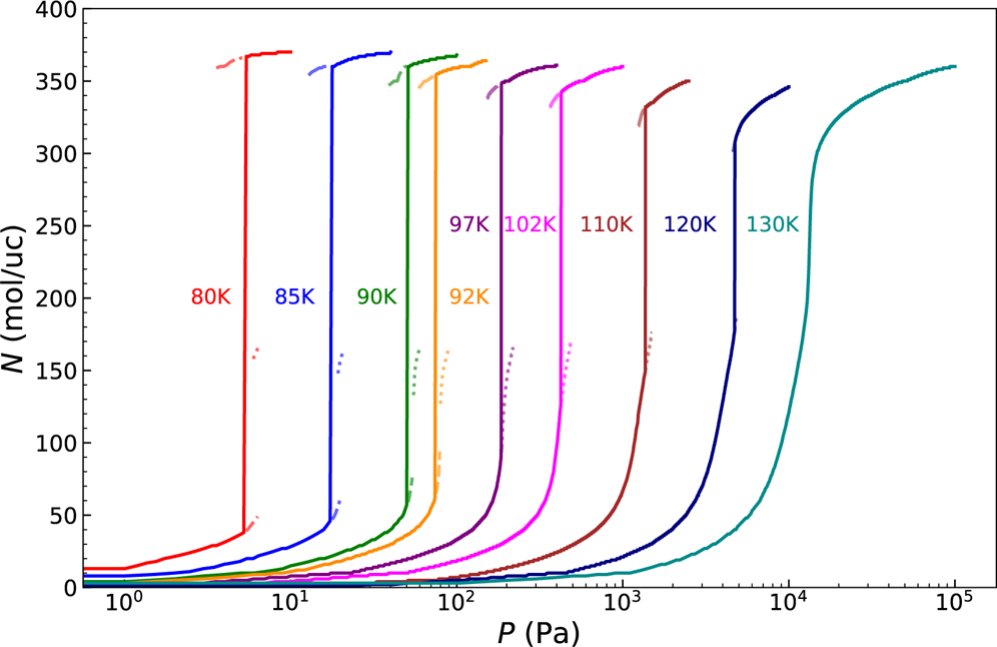
CH_4_ adsorption isotherms from TMMC simulations at *T* = 80–130 K. Discontinuities decrease with increasing temperature. Pressure is plotted on a logarithmic scale.

Figure 4a-c compares adsorption/desorption isotherms from GCMC simulations with TMMC isotherms (including the metastable states) at *T* = 80, 110, and 130 K. Significant hysteresis is observed at low temperatures and diminishes as temperature rises. For *T* ≳ 100 K, GCMC and TMMC isotherms converge, consistent with the vanishing of free energy barriers, as shown in Figure 4d,e: hysteresis is expected only when *W*_b_ ≫ *k*_B_ *T*. By 130 K the discontinuity in *N* disappears.

**Figure 4:**
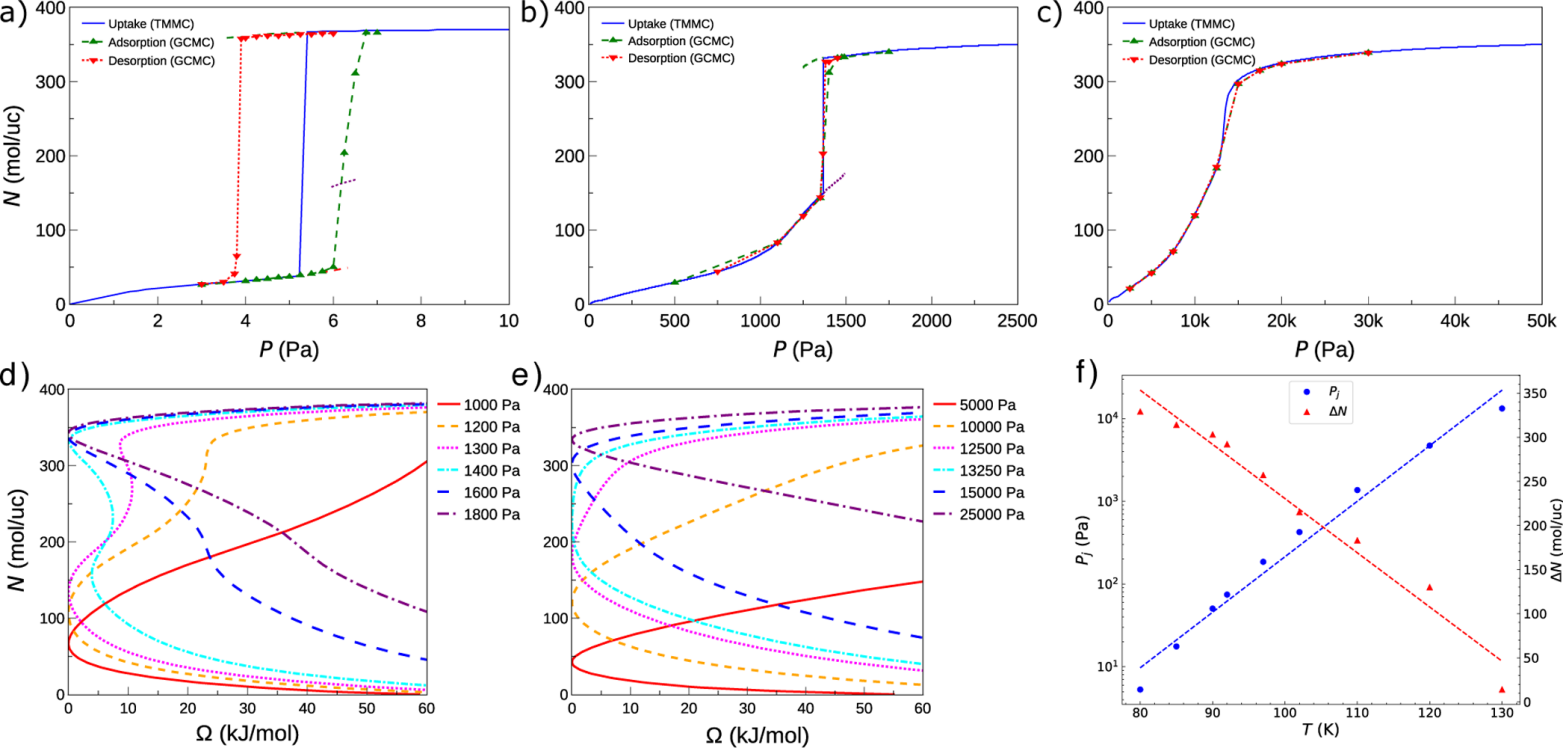
Comparison of GCMC and TMMC isotherms for *T* = 80 K (a), 110 K (b), and 130 K (c). Pronounced hysteresis seen for GCMC curves at low *T* diminishes with increasing temperature. TMMC free energy profiles Ω­(*N*) at *T* = 110 K (d) and 130 K (e). (f) Blue circles: temperature dependence of the transition pressure *P*_j_ (where the adsorption uptake *N* increases sharply); red triangles: temperature dependence of the uptake jump Δ*N*.

At lower temperatures, the free energy minimum shifts discontinuously with pressure, producing abrupt transitions in occupancy (Figure 2a,c and 4d). In contrast, at intermediate and higher temperatures the global minimum evolves more gradually, resulting in smoother isotherms (Figure 4d,e). Figure 4f illustrates how the jump size Δ*N* decreases linearly with increasing temperature, while the transition pressure *P*_j_ increases exponentially. (This trend is consistent with a linear increase of the chemical potential μ_j_ with *T*, reflecting the logarithmic pressure dependence of μ.)

To explore structural changes during these transitions, density maps were generated from TMMC simulations at 80 K for fixed *N* = 35, 150, and 365 (Figure 5a), i.e., low-, mid-, and high-density conditions. These maps reveal a distinct reorganization of CH_4_ across the framework as loading increases. To quantify this evolution, five regions were identified (Figure 5b), and the number of molecules in each was accumulated through the simulation runs (“counts” in Figure 5c). (Note that each of the regions marked represent one of *X* equivalent sites, as defined by the symmetry of the system, i.e., there are 8 equivalent regions 1, 2, 4, and 5, and 2 equivalent areas for 3.)

**Figure 5:**
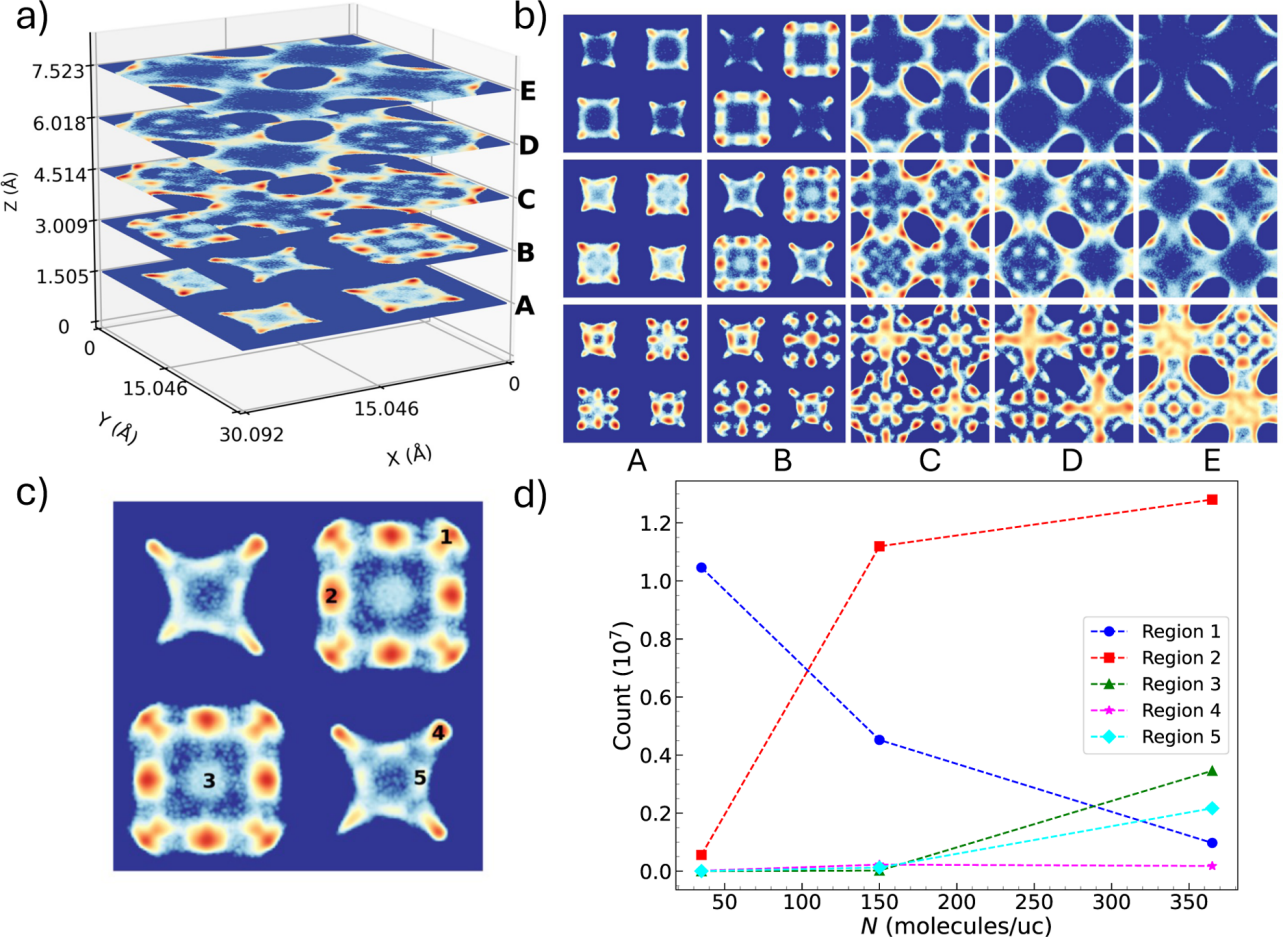
(a) CH_4_ density maps at *T* = 80 K, *N* = 150 in 5 different framework planes (A–E). (b) Density maps at *T* = 80 K for planes A–E, for *N* = 35 (low-density, top panel), 150 (mid-density, middle row), and 365 (high density, bottom row). (c) Designated regions for calculated distribution. (d) Number of molecules accumulated per region as a function of uptake *N*.

As *N* increases (4.29-fold and 2.43-fold in the first and second steps, respectively), the following changes are observed: (i) region 1 depopulates significantly, (ii) region 2 fills rapidly, then gradually, (iii) region 4 remains relatively constant, and (iv) regions 3 and 5 populate only after a threshold at *N* ≈ 150. These trends suggest a structural transition governed by competition between adsorbate–adsorbate and adsorbate–framework interactions.^36^ Initially, low-energy sites are preferentially occupied, but at higher loading, cooperative effects promote the occupation of previously unfilled regions, consistent with the mechanisms on cooperative adsorption phenomena proposed by Mazur et al.^12^

## 4 Summary and Conclusions

In this work, we investigated methane adsorption in a rigid IRMOF-8 model under subcritical conditions using Grand Canonical Monte Carlo (GCMC) and Transition-Matrix Monte Carlo (TMMC) simulations. TMMC provides a statistically rigorous free-energy landscape Ω­(*N*) that reveals multiple minima corresponding to low, intermediate (metastable), and high adsorption states; the intermediate state is associated with window occupation and cooperative adsorbate rearrangement; sharp transitions in the uptake. Our results reveal that adsorption proceeds via discrete transitions between low-, mid-, and high-density states, corresponding to the distinct minima. Notably, we identify a previously unreported mid-density metastable state that emerges in a narrow pressure window and plays a critical role in the observed hysteresis and transition dynamics. This state may be difficult to observe in standard GCMC simulations due to high free energy barriers and slow sampling. Analysis of spatial density distributions demonstrated that these metastable states correspond to distinct structural arrangements of the adsorbed phase, with transitions driven by competing adsorbate–adsorbate and adsorbate–framework interactions. As temperature increased, energy barriers between local minima disappeared, resulting in a smooth uptake transition and suppression of structural rearrangements. This confirms that the observed discontinuities are a cooperative phenomenon that vanishes when thermal energy dominates interaction-driven ordering.

Our results extend previous experimental and computational findings on IRMOF-1 and related frameworks^14,19,20,37–41^ by providing an unbiased, quantitative free-energy reconstruction of adsorption states in IRMOF-8. Compared with biased methods (umbrella sampling, metadynamics), TMMC reconstructs Ω­(*N*) from canonical transition statistics without introducing bias potentials; this provides clearer thermodynamic interpretation of metastability and hysteresis. Our findings are consistent with reports that cooperative rearrangements and adsorption steps in MOFs reflect confined fluid phase behavior rather than solely framework response.^14,20,21,37–39,42^

Experimental verification of the existence of metastable states and cooperative transitions may be difficult due to the adsorbate being inside a solid structure. Techniques such as powder X-ray diffraction (PXRD) or neutron scattering could be used to directly probe the structural rearrangement of the adsorbed methane phase^43–46^ : a sharp, nonlinear change in the Bragg peak intensity or diffuse scattering pattern as pressure is swept across the transition point could provide evidence for the cooperative structural change.

To our knowledge, this is the first quantitative characterization of the complete free energy landscape for methane adsorption in IRMOF-8 and its connection to metastability and cooperative structural changes. Because the model employs a rigid IRMOF-8 framework, it is possible that some of the observed features may not be present in an actual system; like any model it has shortcomings. However, our focus here is the existence of the behavior, which may transcend the actual model limitations and be interesting on their own. Here our findings highlight the critical role of enhanced sampling techniques such as TMMC in revealing metastable adsorption states and phase transitions that may be inaccessible to conventional methods, e.g., for fluids in nanoporous materials. In systems where equilibrium properties depend sensitively on metastability and kinetic barriers, traditional GCMC may yield misleading or incomplete thermodynamic information. Our results provide mechanistic insight beyond conventional adsorption isotherms, revealing energy barriers and cooperative transitions that are inaccessible to standard GCMC approaches.

## Supplementary Material



## Notes

Details on simulation methodology used in this work including RASPA2 configuration files for GCMC and TMMC simulations, additional simulation results of free energies, isotherms, free energy profiles, fluctuations of the uptake during the “desorption cycle” (PDF: IRMOF-8+CH_4_ -Supporting Information.pdf) (PDF)
