# Supplementary material for: Free Energy Landscapes and Metastability in Methane Adsorption within a Representative Metal–Organic Framework
Source: ACS Omega. 2025 Dec 17;10(51):63044–50. doi: 10.1021/acsomega.5c09062 (PMC12756801; doi:10.1021/acsomega.5c09062)
Supplement: Supplementary file 1 [file ao5c09062_si_001.pdf]

# Free Energy Landscapes and Metastability in Methane Adsorption within a Representative Metal-Organic Framework

Anthony Dorhauer[1,2], Malgorzata Stankiewicz[3,4], Bartosz Mazur[4],
Bogdan Kuchta[4,5,*], and Carlos Wexler[1,2,*]

[1]*Deptartment of Physics & Astronomy, University of Missouri, USA*
[2]*Materials Science & Engineering Institute, University of Missouri, USA*
[3]*Faculty of Chemistry, Wroclaw University of Science and Technology, Wybrzeze Wyspianskiego 27, 50-370 Wroclaw, Poland*
[4]*Department of Micro, Nano, and Bioprocess Engineering, 50-370 Wroclaw University of Science and Technology, Poland*
[5]*MADIREL, Aix-Marseille University, 13013 Marseille, France*
**e-mail: wexlerc@missouri.edu, bogdan.kuchta@pwr.edu.pl*

# SUPPLEMENTAL INFORMATION

1. Page 2: Transition Matrix Monte Carlo (TMMC) Free Energies calculated for $T$ = 80, 85, 92, 97, 102, 110, 120, and 130 K, see main manuscript **Equations 3-5**, and **Figures 2ac**, **4de**.

2. Page 5: TMMC Isotherms and Free Energy Profiles calculated for $T$ = 80, 85, 92, 97, 102, 110, 120, and 130 K, see main manuscript **Figures 2abc, 3, 4abcde**.

3. Page 6: Fluctuations in the uptake $N$ during desorption branch in grand canonical Monte Carlo (GCMC) simulations starting from a $N$ = 370 (i.e., high uptake), see main manuscript, **Figure 2defg**.

4. Page 7: Configuration files for RASPA2 GCMC and TMMC simulations:
    a. Example *simulation.input* file for GCMC,
    b. Example *simulation.input* file for TMMC,
    c. Example *simulation.input* file for Canonical Monte Carlo density calculations,
    d. Example *methane.def* file,
    e. Example *pseudo_atoms.def* file,
    f. Example *force_field_mixing_rules.def* file,
    g. Example *IRMOF-8.cif* file.

## 1. TMMC Free Energies

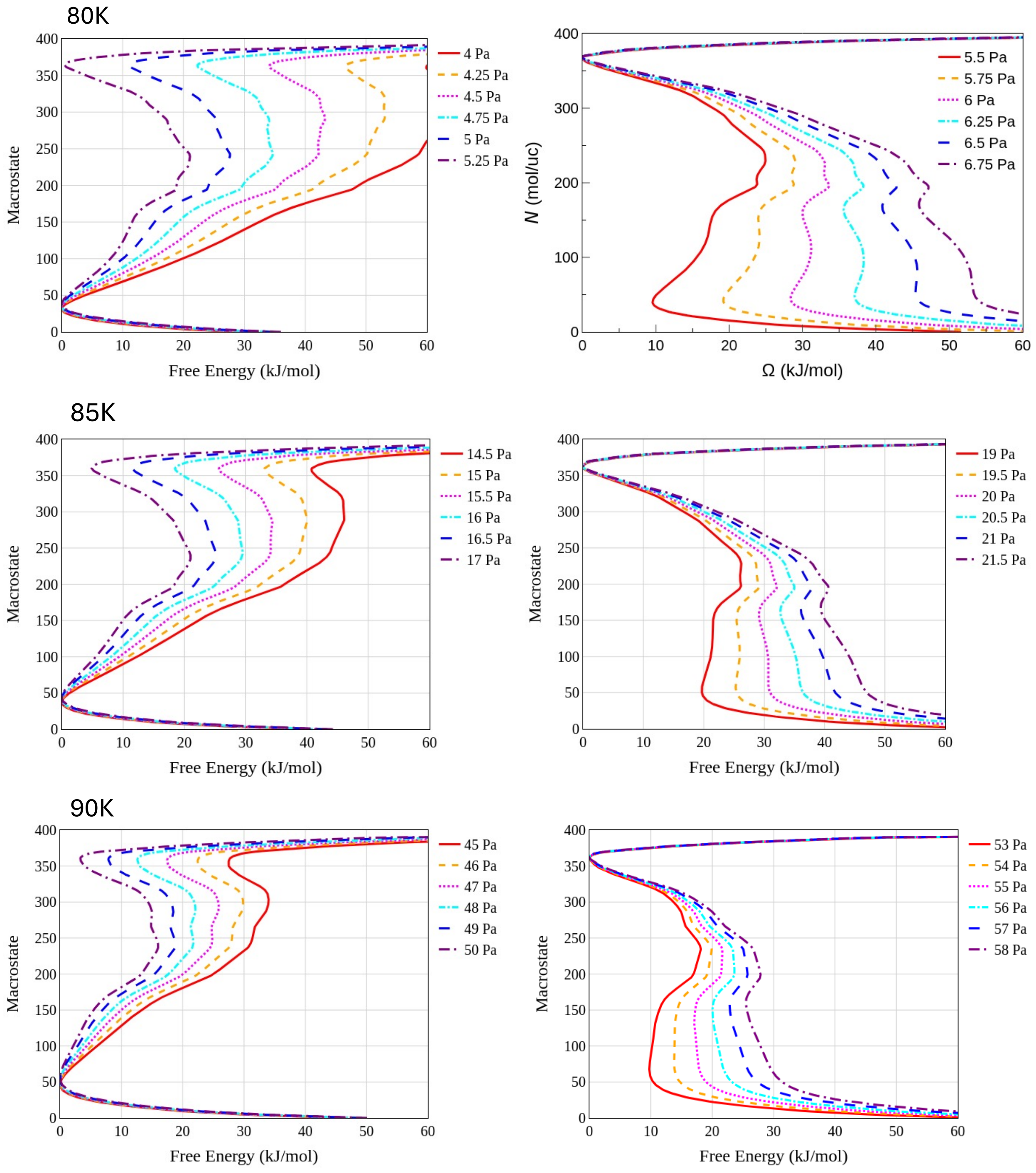


**Figure S1a.** TMMC free energies $\Omega(N)$ for $T = 80$, 85, and 90 K.

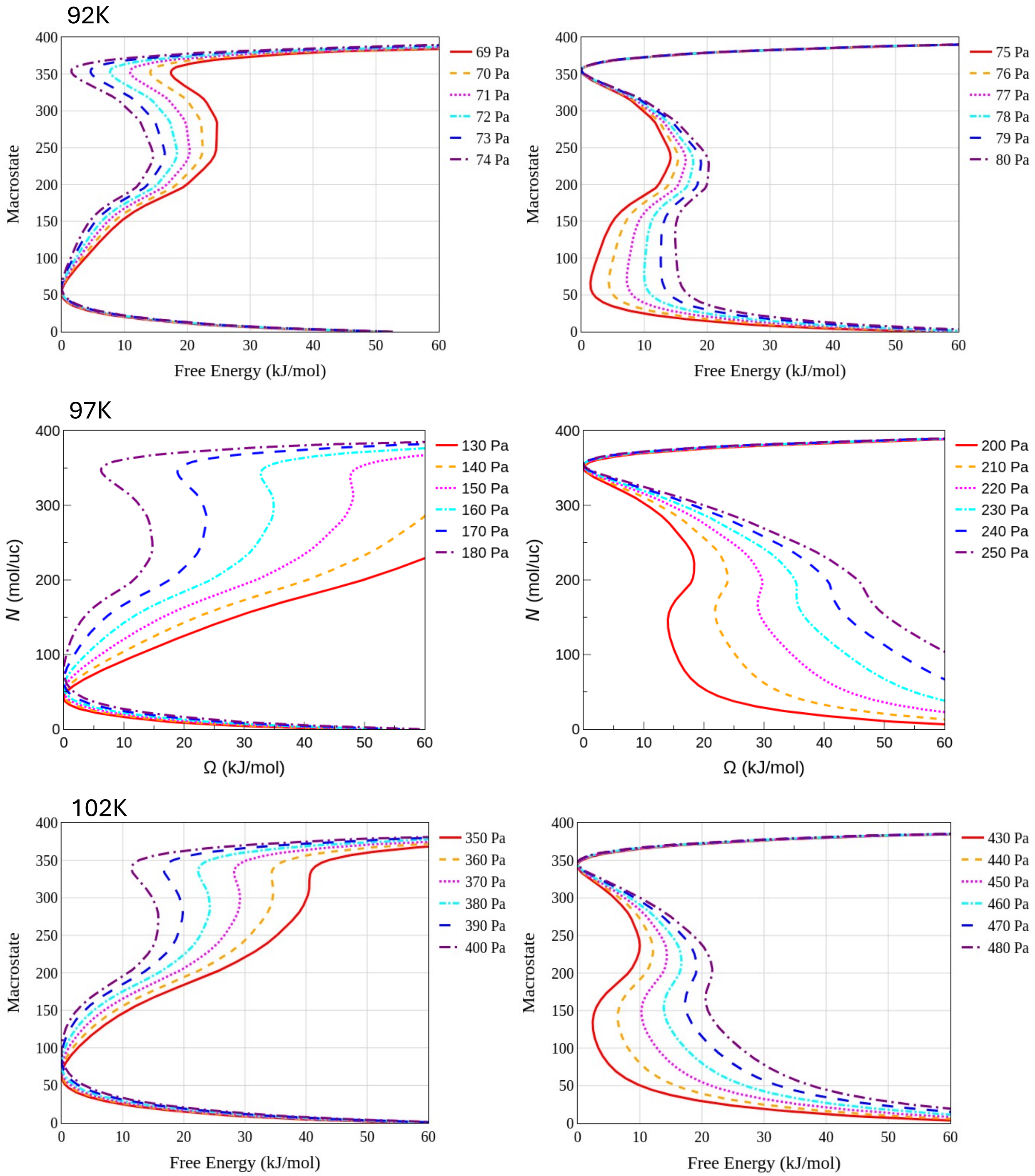


**Figure S1b.** TMMC free energies $\Omega(N)$ for $T = 92, 97, 102$ K.

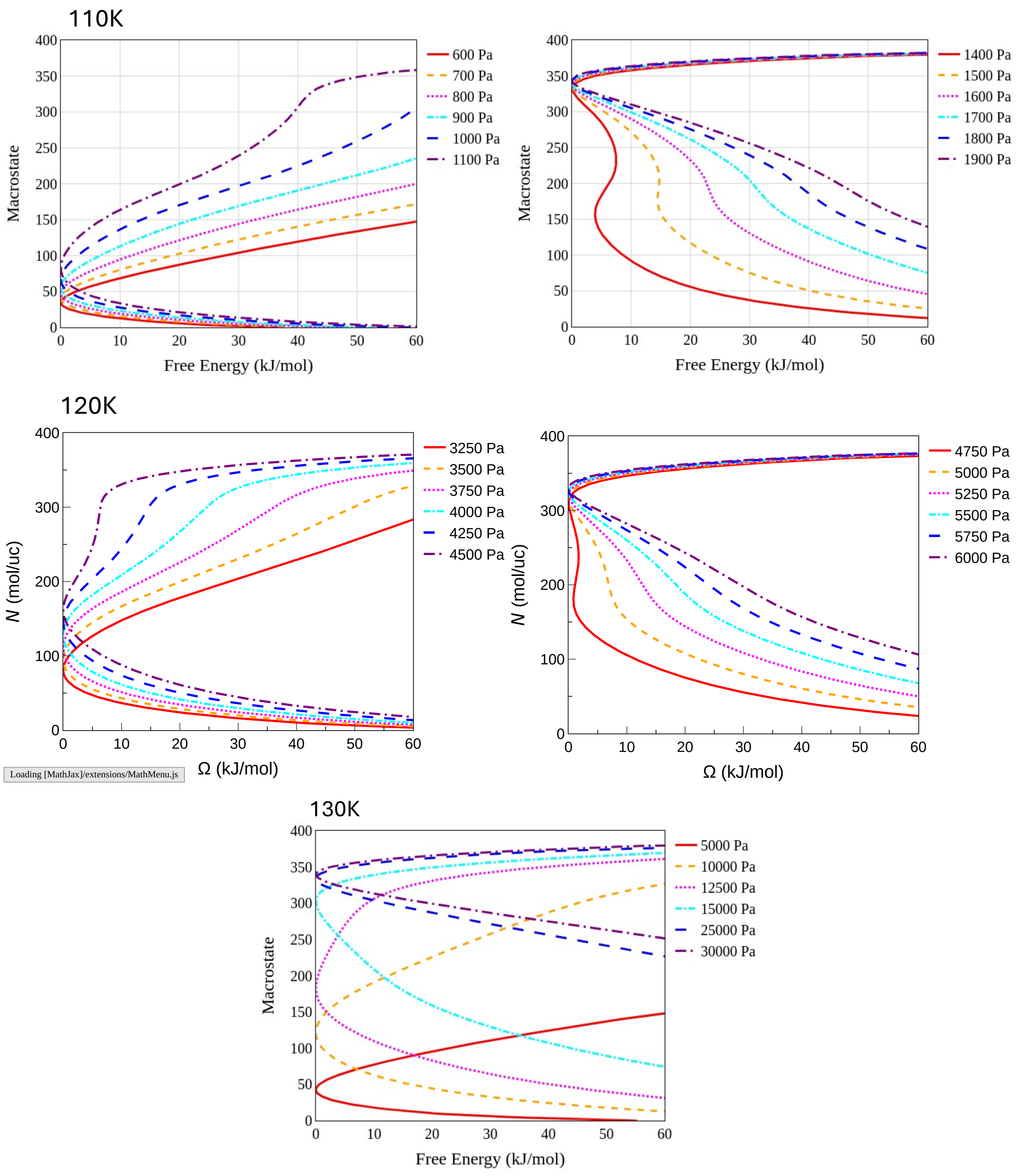


**Figure S1c.** TMMC free energies $\Omega(N)$ for $T = 110, 120, 130$ K.

## 2. TMMC Isotherms and Free Energy Profiles

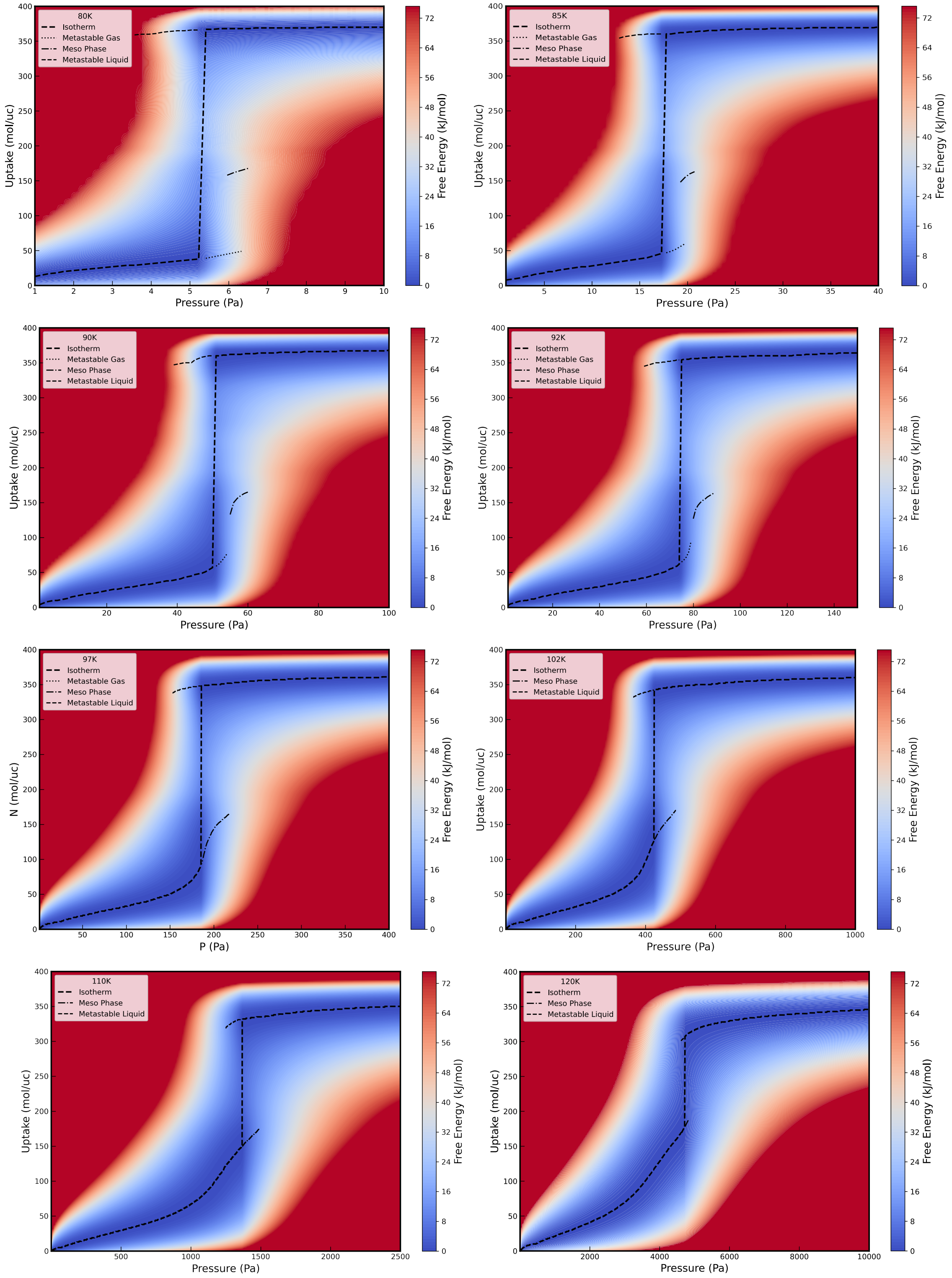


**Figure S2a.** TMMC Isotherms and Free Energy profiles (as density plots) for $T$ = 80, 85, 90, 92, 97, 102, 110, 120 K.

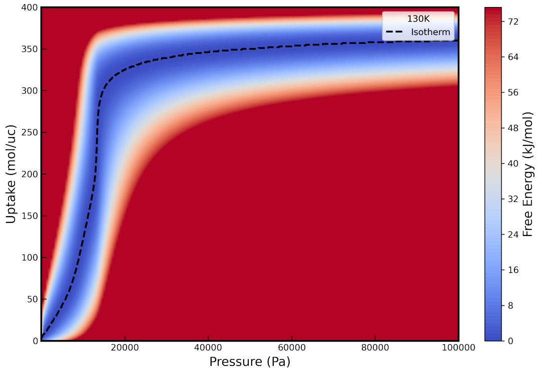


**Figure S2b.** TMMC Isotherms and Free Energy profiles (as density plots) for $T$ = 130 K.

## 3. Fluctuations in the uptake *N* during desorption branch in grand canonical Monte Carlo simulations starting from a *N* = 370 (i.e., high uptake)

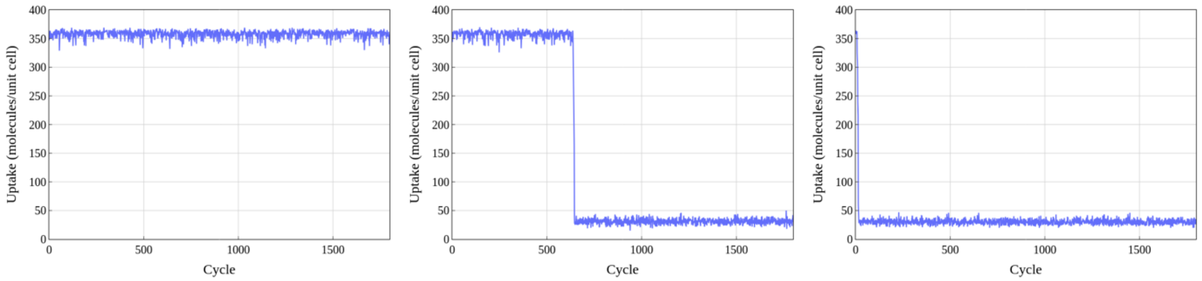


**Figure S3.** Fluctuations in the uptake $N$ during desorption branch GCMC simulations starting from at $T$ = 80 K, $P$ = 3.90 (left), 3.85 (center), and 3.80 Pa (right). For all of these pressures the true equilibrium condition corresponds to a small $N$.

## 4. Configuration files for RASPA2 GCMC and TMMC simulations

### 4.a – Example *simulation.input* file for GCMC

```
SimulationType                      MonteCarlo
NumberOfCycles                      2000000
NumberOfInitializationCycles        200000
PrintEvery                          1000
RestartFile                         no
ContinueAfterCrash                  yes
WriteBinaryRestartFileEvery   1000
Movies                              yes
WriteMoviesEvery              1000
CutOffVDW                           14.0

Framework                           0
FrameworkName                       IRMOF-8
UnitCells                           1 1 1
RemoveAtomNumberCodeFromLabel       yes
UseChargesFromCIFFile         no
ChargeMethod                        None

ExternalTemperature                 80
ExternalPressure              6.25

Component 0 MoleculeName            methane
FugacityCoefficient                 1.0

TranslationProbability              1.0
ReinsertionProbability              1.0
SwapProbability                     1.0
CreateNumberOfMolecules             0
```

### 4.b – Example *simulation.input* file for TMMC

```
SimulationType                      MonteCarlo
NumberOfCycles                      1000000
NumberOfInitializationCycles        50000
PrintEvery                          1000
RestartFile                         no
ContinueAfterCrash                  no
WriteBinaryRestartFileEvery         0
Movies                              no
CutOffVDW                           14.0

RandomSeed 7

Framework                     0
FrameworkName                 IRMOF-8
UnitCells                     1 1 1
RemoveAtomNumberCodeFromLabel       yes
UseChargesFromCIFFile         no
ChargeMethod                        None

ExternalTemperature           80
```

```
ExternalPressure              6.25

PrintGhostProbabilitesEvery   1

Component 0 MoleculeName            methane
FugacityCoefficient                 1.0

TranslationProbability              1.0
RotationProbability                 1.0
ReinsertionProbability              1.0
GhostSwapProbability                1.0
CreateNumberOfMolecules             400
```

## 4.c – Example *simulation.input* file for Canonical Monte Carlo density calculations

```
SimulationType                      MonteCarlo
NumberOfCycles                      2000000
NumberOfInitializationCycles        10000
PrintEvery                          1000
RestartFile                         no
ContinueAfterCrash                  no
WriteBinaryRestartFileEvery         0
Movies                              no
CutOffVDW 14.0

Framework                           0
FrameworkName                       IRMOF-8
UnitCells                           1 1 1
RemoveAtomNumberCodeFromLabel       yes
UseChargesFromCIFFile         no
ChargeMethod                        None

ComputeDensityProfile3DVTKGrid      yes
WriteDensityProfile3DVTKGridEvery 1000
DensityProfile3DVTKGridPoints       150 150 150
NumberOfGrids                       1
GridTypes                           CH4
SpacingVDWGrid                      0.05
UseTabularGrid                      yes

ExternalTemperature                 80
ExternalPressure              6.25

Component 0 MoleculeName            methane

TranslationProbability              1.0
ReinsertionProbability              1.0
SwapProbability                     0
CreateNumberOfMolecules             150
```

## 4.d – Example *methane.def* file

```
# critical constants: Temp. [T], Pressure [Pa], and Acentric factor [-]
190.564
4599200.0
0.01142
# Number Of Atoms
```

```
1
# Number Of Groups
1
# Alkane-group
flexible
# number of atoms
1
# atomic positions
0 CH4
# Chiral centers Bond  BondDipoles Bend  UrayBradley InvBend  Torsion Imp.
Torsion Bond/Bond Bond/Bend Bend/Bend Bond/Torsion Bend/Torsion IntraVDW Intra
ch-ch Intra ch-bd Intra bd-bd
0     0     0     0     0     0     0     0     0     0     0     0     0
      0     0     0     0
# Number of config moves
0
```

4.e – Example *pseudo_atoms.def* file

```
# number of pseudo atoms
1
# type    print   as    chem  oxidation   mass       charge   polarization B-
factor radii  connectivity anisotropic anisotropic-type   tinker-type
CH4   yes   C     C     0     16.04246    0.0   0.0   1.0   1.00  0     0
      relative    0
```

4.f – Example *force_field_mixing_rules.def* file

```
# general rule for shifted vs truncated
shifted
# general rule tailcorrections
no
# number of defined interactions
7
# type interaction
Zn    LENNARD_JONES     62.3996    2.4616   // UFF
O     LENNARD_JONES     48.1584    3.0332   // Dreiding
C     LENNARD_JONES     47.8565    3.4730   // Dreiding
H     LENNARD_JONES      7.6490    2.8464   // Dreiding
CH4   LENNARD_JONES    158.5       3.65     // DACNIS modified
C_co2 LENNARD_JONES     27.0       2.8      // TraPPE
O_co2 LENNARD_JONES     79.0       3.05     // TraPPE
# general mixing rule for Lennard-Jones
Lorentz-Berthelot
```

4.g – Example *IRMOF-8.cif* file

```
data_image0
_cell_length_a       30.0915
_cell_length_b       30.0915
_cell_length_c       30.0915
_cell_angle_alpha    90
_cell_angle_beta     90
_cell_angle_gamma    90
```

```
_symmetry_space_group_name_H-M    "P 1"
_symmetry_int_tables_number       1

loop_
  _symmetry_equiv_pos_as_xyz
  'x, y, z'

loop_
  _atom_site_label
  _atom_site_occupancy
  _atom_site_fract_x
  _atom_site_fract_y
  _atom_site_fract_z
  _atom_site_thermal_displace_type
  _atom_site_B_iso_or_equiv
  _atom_site_type_symbol
  _atom_site_charge
  Zn1      1.0000 0.21282  0.28718  0.21282  Biso   1.000  Zn  0.9782780665372923
  O1       1.0000 0.25000  0.25000  0.25000  Biso   1.000  O  -1.0468075009829967
  O2       1.0000 0.27541  0.65231  0.27541  Biso   1.000  O  -0.5642717423774206
  C1       1.0000 0.25000  0.63255  0.25000  Biso   1.000  C  0.6168858662624891
  C2       1.0000 0.25000  0.58329  0.25000  Biso   1.000  C  -0.0339681943383726
  C3       1.0000 0.27297  0.55331  0.27297  Biso   1.000  C  -0.1379813076941497
  Zn2      1.0000 0.78718  0.71282  0.21282  Biso   1.000  Zn  0.9782780665372923
  O3       1.0000 0.75000  0.75000  0.25000  Biso   1.000  O  -1.0468075009829967
  O4       1.0000 0.72459  0.34769  0.27541  Biso   1.000  O  -0.5642717423774206
  C4       1.0000 0.75000  0.36745  0.25000  Biso   1.000  C  0.6168858662624891
  C5       1.0000 0.75000  0.41671  0.25000  Biso   1.000  C  -0.0339681943383726
  C6       1.0000 0.71804  0.43012  0.28196  Biso   1.000  C  -0.1194810032453188
  C7       1.0000 0.70945  0.47517  0.29055  Biso   1.000  C  -0.1194810032453188
  C8       1.0000 0.73406  0.50700  0.26594  Biso   1.000  C  0.0889764207647183
  Zn3      1.0000 0.78718  0.28718  0.78718  Biso   1.000  Zn  0.9782780665372923
  O5       1.0000 0.75000  0.25000  0.75000  Biso   1.000  O  -1.0468075009829967
  O6       1.0000 0.72459  0.65231  0.72459  Biso   1.000  O  -0.5642717423774206
  C9       1.0000 0.75000  0.63255  0.75000  Biso   1.000  C  0.6168858662624891
  C10      1.0000 0.75000  0.58329  0.75000  Biso   1.000  C  -0.0339681943383726
  C11      1.0000 0.72703  0.55331  0.72703  Biso   1.000  C  -0.1379813076941497
  Zn4      1.0000 0.21282  0.71282  0.78718  Biso   1.000  Zn  0.9782780665372923
  O7       1.0000 0.25000  0.75000  0.75000  Biso   1.000  O  -1.0468075009829967
  O8       1.0000 0.27541  0.34769  0.72459  Biso   1.000  O  -0.5642717423774206
  C12      1.0000 0.25000  0.36745  0.75000  Biso   1.000  C  0.6168858662624891
  C13      1.0000 0.25000  0.41671  0.75000  Biso   1.000  C  -0.0339681943383726
  C14      1.0000 0.29055  0.47517  0.70945  Biso   1.000  C  -0.1194810032453188
  C15      1.0000 0.26594  0.50700  0.73406  Biso   1.000  C  0.0889764207647183
  Zn5      1.0000 0.21282  0.21282  0.28718  Biso   1.000  Zn  0.9782780665372923
  O9       1.0000 0.27541  0.27541  0.65231  Biso   1.000  O  -0.5642717423774206
  C16      1.0000 0.25000  0.25000  0.63255  Biso   1.000  C  0.6168858662624891
  C17      1.0000 0.25000  0.25000  0.58329  Biso   1.000  C  -0.0339681943383726
  C18      1.0000 0.28196  0.28196  0.56988  Biso   1.000  C  -0.1194810032453188
  C19      1.0000 0.29055  0.29055  0.52483  Biso   1.000  C  -0.1194810032453188
  C20      1.0000 0.26594  0.26594  0.49300  Biso   1.000  C  0.0889764207647183
  Zn6      1.0000 0.21282  0.78718  0.71282  Biso   1.000  Zn  0.9782780665372923
  O10      1.0000 0.27541  0.72459  0.34769  Biso   1.000  O  -0.5642717423774206
  C21      1.0000 0.25000  0.75000  0.36745  Biso   1.000  C  0.6168858662624891
  C22      1.0000 0.25000  0.75000  0.41671  Biso   1.000  C  -0.0339681943383726
  C23      1.0000 0.27297  0.72703  0.44669  Biso   1.000  C  -0.1379813076941497
  Zn7      1.0000 0.78718  0.78718  0.28718  Biso   1.000  Zn  0.9782780665372923
  O11      1.0000 0.72459  0.72459  0.65231  Biso   1.000  O  -0.5642717423774206
```

| | | | | | | | | |
|---|---|---|---|---|---|---|---|---|
| C24 | 1.0000 | 0.75000 | 0.75000 | 0.63255 | Biso | 1.000 | C | 0.6168858662624891 |
| C25 | 1.0000 | 0.75000 | 0.75000 | 0.58329 | Biso | 1.000 | C | -0.0339681943383726 |
| C26 | 1.0000 | 0.71804 | 0.71804 | 0.56988 | Biso | 1.000 | C | -0.1194810032453188 |
| C27 | 1.0000 | 0.70945 | 0.70945 | 0.52483 | Biso | 1.000 | C | -0.1194810032453188 |
| C28 | 1.0000 | 0.73406 | 0.73406 | 0.49300 | Biso | 1.000 | C | 0.0889764207647183 |
| Zn8 | 1.0000 | 0.78718 | 0.21282 | 0.71282 | Biso | 1.000 | Zn | 0.9782780665372923 |
| O12 | 1.0000 | 0.72459 | 0.27541 | 0.34769 | Biso | 1.000 | O | -0.5642717423774206 |
| C29 | 1.0000 | 0.75000 | 0.25000 | 0.36745 | Biso | 1.000 | C | 0.6168858662624891 |
| C30 | 1.0000 | 0.75000 | 0.25000 | 0.41671 | Biso | 1.000 | C | -0.0339681943383726 |
| C31 | 1.0000 | 0.72703 | 0.27297 | 0.44669 | Biso | 1.000 | C | -0.1379813076941497 |
| Zn9 | 1.0000 | 0.28718 | 0.21282 | 0.21282 | Biso | 1.000 | Zn | 0.9782780665372923 |
| O13 | 1.0000 | 0.65231 | 0.27541 | 0.27541 | Biso | 1.000 | O | -0.5642717423774206 |
| C32 | 1.0000 | 0.63255 | 0.25000 | 0.25000 | Biso | 1.000 | C | 0.6168858662624891 |
| C33 | 1.0000 | 0.58329 | 0.25000 | 0.25000 | Biso | 1.000 | C | -0.0339681943383726 |
| C34 | 1.0000 | 0.56988 | 0.28196 | 0.28196 | Biso | 1.000 | C | -0.1194810032453188 |
| C35 | 1.0000 | 0.52483 | 0.29055 | 0.29055 | Biso | 1.000 | C | -0.1194810032453188 |
| C36 | 1.0000 | 0.49300 | 0.26594 | 0.26594 | Biso | 1.000 | C | 0.0889764207647183 |
| Zn10 | 1.0000 | 0.71282 | 0.21282 | 0.78718 | Biso | 1.000 | Zn | 0.9782780665372923 |
| O14 | 1.0000 | 0.34769 | 0.27541 | 0.72459 | Biso | 1.000 | O | -0.5642717423774206 |
| C37 | 1.0000 | 0.36745 | 0.25000 | 0.75000 | Biso | 1.000 | C | 0.6168858662624891 |
| C38 | 1.0000 | 0.41671 | 0.25000 | 0.75000 | Biso | 1.000 | C | -0.0339681943383726 |
| C39 | 1.0000 | 0.44669 | 0.27297 | 0.72703 | Biso | 1.000 | C | -0.1379813076941497 |
| Zn11 | 1.0000 | 0.28718 | 0.78718 | 0.78718 | Biso | 1.000 | Zn | 0.9782780665372923 |
| O15 | 1.0000 | 0.65231 | 0.72459 | 0.72459 | Biso | 1.000 | O | -0.5642717423774206 |
| C40 | 1.0000 | 0.63255 | 0.75000 | 0.75000 | Biso | 1.000 | C | 0.6168858662624891 |
| C41 | 1.0000 | 0.58329 | 0.75000 | 0.75000 | Biso | 1.000 | C | -0.0339681943383726 |
| C42 | 1.0000 | 0.56988 | 0.71804 | 0.71804 | Biso | 1.000 | C | -0.1194810032453188 |
| C43 | 1.0000 | 0.52483 | 0.70945 | 0.70945 | Biso | 1.000 | C | -0.1194810032453188 |
| C44 | 1.0000 | 0.49300 | 0.73406 | 0.73406 | Biso | 1.000 | C | 0.0889764207647183 |
| Zn12 | 1.0000 | 0.71282 | 0.78718 | 0.21282 | Biso | 1.000 | Zn | 0.9782780665372923 |
| O16 | 1.0000 | 0.34769 | 0.72459 | 0.27541 | Biso | 1.000 | O | -0.5642717423774206 |
| C45 | 1.0000 | 0.36745 | 0.75000 | 0.25000 | Biso | 1.000 | C | 0.6168858662624891 |
| C46 | 1.0000 | 0.41671 | 0.75000 | 0.25000 | Biso | 1.000 | C | -0.0339681943383726 |
| C47 | 1.0000 | 0.44669 | 0.72703 | 0.27297 | Biso | 1.000 | C | -0.1379813076941497 |
| Zn13 | 1.0000 | 0.28718 | 0.21282 | 0.78718 | Biso | 1.000 | Zn | 0.9782780665372923 |
| O17 | 1.0000 | 0.25000 | 0.25000 | 0.75000 | Biso | 1.000 | O | -1.0468075009829967 |
| O18 | 1.0000 | 0.65231 | 0.27541 | 0.72459 | Biso | 1.000 | O | -0.5642717423774206 |
| C48 | 1.0000 | 0.63255 | 0.25000 | 0.75000 | Biso | 1.000 | C | 0.6168858662624891 |
| C49 | 1.0000 | 0.58329 | 0.25000 | 0.75000 | Biso | 1.000 | C | -0.0339681943383726 |
| C50 | 1.0000 | 0.56988 | 0.28196 | 0.71804 | Biso | 1.000 | C | -0.1194810032453188 |
| C51 | 1.0000 | 0.52483 | 0.29055 | 0.70945 | Biso | 1.000 | C | -0.1194810032453188 |
| C52 | 1.0000 | 0.49300 | 0.26594 | 0.73406 | Biso | 1.000 | C | 0.0889764207647183 |
| Zn14 | 1.0000 | 0.71282 | 0.78718 | 0.78718 | Biso | 1.000 | Zn | 0.9782780665372923 |
| O19 | 1.0000 | 0.75000 | 0.75000 | 0.75000 | Biso | 1.000 | O | -1.0468075009829967 |
| O20 | 1.0000 | 0.34769 | 0.72459 | 0.72459 | Biso | 1.000 | O | -0.5642717423774206 |
| C53 | 1.0000 | 0.36745 | 0.75000 | 0.75000 | Biso | 1.000 | C | 0.6168858662624891 |
| C54 | 1.0000 | 0.41671 | 0.75000 | 0.75000 | Biso | 1.000 | C | -0.0339681943383726 |
| C55 | 1.0000 | 0.44669 | 0.72703 | 0.72703 | Biso | 1.000 | C | -0.1379813076941497 |
| Zn15 | 1.0000 | 0.28718 | 0.78718 | 0.21282 | Biso | 1.000 | Zn | 0.9782780665372923 |
| O21 | 1.0000 | 0.25000 | 0.75000 | 0.25000 | Biso | 1.000 | O | -1.0468075009829967 |
| O22 | 1.0000 | 0.65231 | 0.72459 | 0.27541 | Biso | 1.000 | O | -0.5642717423774206 |
| C56 | 1.0000 | 0.63255 | 0.75000 | 0.25000 | Biso | 1.000 | C | 0.6168858662624891 |
| C57 | 1.0000 | 0.58329 | 0.75000 | 0.25000 | Biso | 1.000 | C | -0.0339681943383726 |
| C58 | 1.0000 | 0.56988 | 0.71804 | 0.28196 | Biso | 1.000 | C | -0.1194810032453188 |
| C59 | 1.0000 | 0.52483 | 0.70945 | 0.29055 | Biso | 1.000 | C | -0.1194810032453188 |
| C60 | 1.0000 | 0.49300 | 0.73406 | 0.26594 | Biso | 1.000 | C | 0.0889764207647183 |
| Zn16 | 1.0000 | 0.71282 | 0.21282 | 0.21282 | Biso | 1.000 | Zn | 0.9782780665372923 |
| O23 | 1.0000 | 0.75000 | 0.25000 | 0.25000 | Biso | 1.000 | O | -1.0468075009829967 |

| | | | | | | | | |
|---|---|---|---|---|---|---|---|---|
| O24 | 1.0000 | 0.34769 | 0.27541 | 0.27541 | Biso | 1.000 | O | -0.5642717423774206 |
| C61 | 1.0000 | 0.36745 | 0.25000 | 0.25000 | Biso | 1.000 | C | 0.6168858662624891 |
| C62 | 1.0000 | 0.41671 | 0.25000 | 0.25000 | Biso | 1.000 | C | -0.0339681943383726 |
| C63 | 1.0000 | 0.44669 | 0.27297 | 0.27297 | Biso | 1.000 | C | -0.1379813076941497 |
| Zn17 | 1.0000 | 0.21282 | 0.21282 | 0.71282 | Biso | 1.000 | Zn | 0.9782780665372923 |
| O25 | 1.0000 | 0.27541 | 0.27541 | 0.34769 | Biso | 1.000 | O | -0.5642717423774206 |
| C64 | 1.0000 | 0.25000 | 0.25000 | 0.36745 | Biso | 1.000 | C | 0.6168858662624891 |
| C65 | 1.0000 | 0.25000 | 0.25000 | 0.41671 | Biso | 1.000 | C | -0.0339681943383726 |
| C66 | 1.0000 | 0.27297 | 0.27297 | 0.44669 | Biso | 1.000 | C | -0.1379813076941497 |
| Zn18 | 1.0000 | 0.78718 | 0.21282 | 0.28718 | Biso | 1.000 | Zn | 0.9782780665372923 |
| O26 | 1.0000 | 0.72459 | 0.27541 | 0.65231 | Biso | 1.000 | O | -0.5642717423774206 |
| C67 | 1.0000 | 0.75000 | 0.25000 | 0.63255 | Biso | 1.000 | C | 0.6168858662624891 |
| C68 | 1.0000 | 0.75000 | 0.25000 | 0.58329 | Biso | 1.000 | C | -0.0339681943383726 |
| C69 | 1.0000 | 0.71804 | 0.28196 | 0.56988 | Biso | 1.000 | C | -0.1194810032453188 |
| C70 | 1.0000 | 0.70945 | 0.29055 | 0.52483 | Biso | 1.000 | C | -0.1194810032453188 |
| C71 | 1.0000 | 0.73406 | 0.26594 | 0.49300 | Biso | 1.000 | C | 0.0889764207647183 |
| Zn19 | 1.0000 | 0.78718 | 0.78718 | 0.71282 | Biso | 1.000 | Zn | 0.9782780665372923 |
| O27 | 1.0000 | 0.72459 | 0.72459 | 0.34769 | Biso | 1.000 | O | -0.5642717423774206 |
| C72 | 1.0000 | 0.75000 | 0.75000 | 0.36745 | Biso | 1.000 | C | 0.6168858662624891 |
| C73 | 1.0000 | 0.75000 | 0.75000 | 0.41671 | Biso | 1.000 | C | -0.0339681943383726 |
| C74 | 1.0000 | 0.72703 | 0.72703 | 0.44669 | Biso | 1.000 | C | -0.1379813076941497 |
| Zn20 | 1.0000 | 0.21282 | 0.78718 | 0.28718 | Biso | 1.000 | Zn | 0.9782780665372923 |
| O28 | 1.0000 | 0.27541 | 0.72459 | 0.65231 | Biso | 1.000 | O | -0.5642717423774206 |
| C75 | 1.0000 | 0.25000 | 0.75000 | 0.63255 | Biso | 1.000 | C | 0.6168858662624891 |
| C76 | 1.0000 | 0.25000 | 0.75000 | 0.58329 | Biso | 1.000 | C | -0.0339681943383726 |
| C77 | 1.0000 | 0.28196 | 0.71804 | 0.56988 | Biso | 1.000 | C | -0.1194810032453188 |
| C78 | 1.0000 | 0.29055 | 0.70945 | 0.52483 | Biso | 1.000 | C | -0.1194810032453188 |
| C79 | 1.0000 | 0.26594 | 0.73406 | 0.49300 | Biso | 1.000 | C | 0.0889764207647183 |
| Zn21 | 1.0000 | 0.21282 | 0.28718 | 0.78718 | Biso | 1.000 | Zn | 0.9782780665372923 |
| O29 | 1.0000 | 0.27541 | 0.65231 | 0.72459 | Biso | 1.000 | O | -0.5642717423774206 |
| C80 | 1.0000 | 0.25000 | 0.63255 | 0.75000 | Biso | 1.000 | C | 0.6168858662624891 |
| C81 | 1.0000 | 0.25000 | 0.58329 | 0.75000 | Biso | 1.000 | C | -0.0339681943383726 |
| Zn22 | 1.0000 | 0.21282 | 0.71282 | 0.21282 | Biso | 1.000 | Zn | 0.9782780665372923 |
| O30 | 1.0000 | 0.27541 | 0.34769 | 0.27541 | Biso | 1.000 | O | -0.5642717423774206 |
| C82 | 1.0000 | 0.25000 | 0.36745 | 0.25000 | Biso | 1.000 | C | 0.6168858662624891 |
| C83 | 1.0000 | 0.25000 | 0.41671 | 0.25000 | Biso | 1.000 | C | -0.0339681943383726 |
| C84 | 1.0000 | 0.28196 | 0.43012 | 0.28196 | Biso | 1.000 | C | -0.1194810032453188 |
| C85 | 1.0000 | 0.29055 | 0.47517 | 0.29055 | Biso | 1.000 | C | -0.1194810032453188 |
| C86 | 1.0000 | 0.26594 | 0.50700 | 0.26594 | Biso | 1.000 | C | 0.0889764207647183 |
| Zn23 | 1.0000 | 0.78718 | 0.28718 | 0.21282 | Biso | 1.000 | Zn | 0.9782780665372923 |
| O31 | 1.0000 | 0.72459 | 0.65231 | 0.27541 | Biso | 1.000 | O | -0.5642717423774206 |
| C87 | 1.0000 | 0.75000 | 0.63255 | 0.25000 | Biso | 1.000 | C | 0.6168858662624891 |
| C88 | 1.0000 | 0.75000 | 0.58329 | 0.25000 | Biso | 1.000 | C | -0.0339681943383726 |
| C89 | 1.0000 | 0.72703 | 0.55331 | 0.27297 | Biso | 1.000 | C | -0.1379813076941497 |
| Zn24 | 1.0000 | 0.78718 | 0.71282 | 0.78718 | Biso | 1.000 | Zn | 0.9782780665372923 |
| O32 | 1.0000 | 0.72459 | 0.34769 | 0.72459 | Biso | 1.000 | O | -0.5642717423774206 |
| C90 | 1.0000 | 0.75000 | 0.36745 | 0.75000 | Biso | 1.000 | C | 0.6168858662624891 |
| C91 | 1.0000 | 0.75000 | 0.41671 | 0.75000 | Biso | 1.000 | C | -0.0339681943383726 |
| C92 | 1.0000 | 0.71804 | 0.43012 | 0.71804 | Biso | 1.000 | C | -0.1194810032453188 |
| C93 | 1.0000 | 0.70945 | 0.47517 | 0.70945 | Biso | 1.000 | C | -0.1194810032453188 |
| C94 | 1.0000 | 0.73406 | 0.50700 | 0.73406 | Biso | 1.000 | C | 0.0889764207647183 |
| O33 | 1.0000 | 0.27541 | 0.15231 | 0.77541 | Biso | 1.000 | O | -0.5642717423774206 |
| C95 | 1.0000 | 0.25000 | 0.13255 | 0.75000 | Biso | 1.000 | C | 0.6168858662624891 |
| C96 | 1.0000 | 0.25000 | 0.08329 | 0.75000 | Biso | 1.000 | C | -0.0339681943383726 |
| C97 | 1.0000 | 0.27297 | 0.05331 | 0.77297 | Biso | 1.000 | C | -0.1379813076941497 |
| O34 | 1.0000 | 0.72459 | 0.84769 | 0.77541 | Biso | 1.000 | O | -0.5642717423774206 |
| C98 | 1.0000 | 0.75000 | 0.86745 | 0.75000 | Biso | 1.000 | C | 0.6168858662624891 |
| C99 | 1.0000 | 0.75000 | 0.91671 | 0.75000 | Biso | 1.000 | C | -0.0339681943383726 |

| | | | | | | | | |
|---|---|---|---|---|---|---|---|---|
| C100 | 1.0000 | 0.71804 | 0.93012 | 0.78196 | Biso | 1.000 | C | -0.1194810032453188 |
| C101 | 1.0000 | 0.70945 | 0.97517 | 0.79055 | Biso | 1.000 | C | -0.1194810032453188 |
| C102 | 1.0000 | 0.73406 | 0.00700 | 0.76594 | Biso | 1.000 | C | 0.0889764207647183 |
| O35 | 1.0000 | 0.72459 | 0.15231 | 0.22459 | Biso | 1.000 | O | -0.5642717423774206 |
| C103 | 1.0000 | 0.75000 | 0.13255 | 0.25000 | Biso | 1.000 | C | 0.6168858662624891 |
| C104 | 1.0000 | 0.75000 | 0.08329 | 0.25000 | Biso | 1.000 | C | -0.0339681943383726 |
| C105 | 1.0000 | 0.72703 | 0.05331 | 0.22703 | Biso | 1.000 | C | -0.1379813076941497 |
| O36 | 1.0000 | 0.27541 | 0.84769 | 0.22459 | Biso | 1.000 | O | -0.5642717423774206 |
| C106 | 1.0000 | 0.25000 | 0.86745 | 0.25000 | Biso | 1.000 | C | 0.6168858662624891 |
| C107 | 1.0000 | 0.25000 | 0.91671 | 0.25000 | Biso | 1.000 | C | -0.0339681943383726 |
| C108 | 1.0000 | 0.28196 | 0.93012 | 0.21804 | Biso | 1.000 | C | -0.1194810032453188 |
| C109 | 1.0000 | 0.29055 | 0.97517 | 0.20945 | Biso | 1.000 | C | -0.1194810032453188 |
| C110 | 1.0000 | 0.26594 | 0.00700 | 0.23406 | Biso | 1.000 | C | 0.0889764207647183 |
| O37 | 1.0000 | 0.27541 | 0.77541 | 0.15231 | Biso | 1.000 | O | -0.5642717423774206 |
| C111 | 1.0000 | 0.25000 | 0.75000 | 0.13255 | Biso | 1.000 | C | 0.6168858662624891 |
| C112 | 1.0000 | 0.25000 | 0.75000 | 0.08329 | Biso | 1.000 | C | -0.0339681943383726 |
| C113 | 1.0000 | 0.28196 | 0.78196 | 0.06988 | Biso | 1.000 | C | -0.1194810032453188 |
| C114 | 1.0000 | 0.29055 | 0.79055 | 0.02483 | Biso | 1.000 | C | -0.1194810032453188 |
| C115 | 1.0000 | 0.26594 | 0.76594 | 0.99300 | Biso | 1.000 | C | 0.0889764207647183 |
| O38 | 1.0000 | 0.27541 | 0.22459 | 0.84769 | Biso | 1.000 | O | -0.5642717423774206 |
| C116 | 1.0000 | 0.25000 | 0.25000 | 0.86745 | Biso | 1.000 | C | 0.6168858662624891 |
| C117 | 1.0000 | 0.25000 | 0.25000 | 0.91671 | Biso | 1.000 | C | -0.0339681943383726 |
| C118 | 1.0000 | 0.27297 | 0.22703 | 0.94669 | Biso | 1.000 | C | -0.1379813076941497 |
| O39 | 1.0000 | 0.72459 | 0.22459 | 0.15231 | Biso | 1.000 | O | -0.5642717423774206 |
| C119 | 1.0000 | 0.75000 | 0.25000 | 0.13255 | Biso | 1.000 | C | 0.6168858662624891 |
| C120 | 1.0000 | 0.75000 | 0.25000 | 0.08329 | Biso | 1.000 | C | -0.0339681943383726 |
| C121 | 1.0000 | 0.70945 | 0.20945 | 0.02483 | Biso | 1.000 | C | -0.1194810032453188 |
| C122 | 1.0000 | 0.73406 | 0.23406 | 0.99300 | Biso | 1.000 | C | 0.0889764207647183 |
| O40 | 1.0000 | 0.72459 | 0.77541 | 0.84769 | Biso | 1.000 | O | -0.5642717423774206 |
| C123 | 1.0000 | 0.75000 | 0.75000 | 0.86745 | Biso | 1.000 | C | 0.6168858662624891 |
| C124 | 1.0000 | 0.75000 | 0.75000 | 0.91671 | Biso | 1.000 | C | -0.0339681943383726 |
| C125 | 1.0000 | 0.72703 | 0.77297 | 0.94669 | Biso | 1.000 | C | -0.1379813076941497 |
| Zn25 | 1.0000 | 0.28718 | 0.71282 | 0.71282 | Biso | 1.000 | Zn | 0.9782780665372923 |
| O41 | 1.0000 | 0.65231 | 0.77541 | 0.77541 | Biso | 1.000 | O | -0.5642717423774206 |
| C126 | 1.0000 | 0.55331 | 0.77297 | 0.77297 | Biso | 1.000 | C | -0.1379813076941497 |
| Zn26 | 1.0000 | 0.71282 | 0.71282 | 0.28718 | Biso | 1.000 | Zn | 0.9782780665372923 |
| O42 | 1.0000 | 0.34769 | 0.77541 | 0.22459 | Biso | 1.000 | O | -0.5642717423774206 |
| C127 | 1.0000 | 0.43012 | 0.78196 | 0.21804 | Biso | 1.000 | C | -0.1194810032453188 |
| C128 | 1.0000 | 0.47517 | 0.79055 | 0.20945 | Biso | 1.000 | C | -0.1194810032453188 |
| C129 | 1.0000 | 0.50700 | 0.76594 | 0.23406 | Biso | 1.000 | C | 0.0889764207647183 |
| Zn27 | 1.0000 | 0.28718 | 0.28718 | 0.28718 | Biso | 1.000 | Zn | 0.9782780665372923 |
| O43 | 1.0000 | 0.65231 | 0.22459 | 0.22459 | Biso | 1.000 | O | -0.5642717423774206 |
| C130 | 1.0000 | 0.55331 | 0.22703 | 0.22703 | Biso | 1.000 | C | -0.1379813076941497 |
| Zn28 | 1.0000 | 0.71282 | 0.28718 | 0.71282 | Biso | 1.000 | Zn | 0.9782780665372923 |
| O44 | 1.0000 | 0.34769 | 0.22459 | 0.77541 | Biso | 1.000 | O | -0.5642717423774206 |
| C131 | 1.0000 | 0.43012 | 0.21804 | 0.78196 | Biso | 1.000 | C | -0.1194810032453188 |
| C132 | 1.0000 | 0.47517 | 0.20945 | 0.79055 | Biso | 1.000 | C | -0.1194810032453188 |
| C133 | 1.0000 | 0.50700 | 0.23406 | 0.76594 | Biso | 1.000 | C | 0.0889764207647183 |
| Zn29 | 1.0000 | 0.28718 | 0.71282 | 0.28718 | Biso | 1.000 | Zn | 0.9782780665372923 |
| O45 | 1.0000 | 0.65231 | 0.77541 | 0.22459 | Biso | 1.000 | O | -0.5642717423774206 |
| C134 | 1.0000 | 0.55331 | 0.77297 | 0.22703 | Biso | 1.000 | C | -0.1379813076941497 |
| Zn30 | 1.0000 | 0.71282 | 0.28718 | 0.28718 | Biso | 1.000 | Zn | 0.9782780665372923 |
| O46 | 1.0000 | 0.34769 | 0.22459 | 0.22459 | Biso | 1.000 | O | -0.5642717423774206 |
| C135 | 1.0000 | 0.43012 | 0.21804 | 0.21804 | Biso | 1.000 | C | -0.1194810032453188 |
| C136 | 1.0000 | 0.47517 | 0.20945 | 0.20945 | Biso | 1.000 | C | -0.1194810032453188 |
| C137 | 1.0000 | 0.50700 | 0.23406 | 0.23406 | Biso | 1.000 | C | 0.0889764207647183 |
| Zn31 | 1.0000 | 0.28718 | 0.28718 | 0.71282 | Biso | 1.000 | Zn | 0.9782780665372923 |
| O47 | 1.0000 | 0.65231 | 0.22459 | 0.77541 | Biso | 1.000 | O | -0.5642717423774206 |

| | | | | | | | | |
|---|---|---|---|---|---|---|---|---|
| C138 | 1.0000 | 0.55331 | 0.22703 | 0.77297 | Biso | 1.000 | C | -0.1379813076941497 |
| Zn32 | 1.0000 | 0.71282 | 0.71282 | 0.71282 | Biso | 1.000 | Zn | 0.9782780665372923 |
| O48 | 1.0000 | 0.34769 | 0.77541 | 0.77541 | Biso | 1.000 | O | -0.5642717423774206 |
| C139 | 1.0000 | 0.43012 | 0.78196 | 0.78196 | Biso | 1.000 | C | -0.1194810032453188 |
| C140 | 1.0000 | 0.47517 | 0.79055 | 0.79055 | Biso | 1.000 | C | -0.1194810032453188 |
| C141 | 1.0000 | 0.50700 | 0.76594 | 0.76594 | Biso | 1.000 | C | 0.0889764207647183 |
| O49 | 1.0000 | 0.27541 | 0.77541 | 0.84769 | Biso | 1.000 | O | -0.5642717423774206 |
| C142 | 1.0000 | 0.25000 | 0.75000 | 0.86745 | Biso | 1.000 | C | 0.6168858662624891 |
| C143 | 1.0000 | 0.25000 | 0.75000 | 0.91671 | Biso | 1.000 | C | -0.0339681943383726 |
| C144 | 1.0000 | 0.27297 | 0.77297 | 0.94669 | Biso | 1.000 | C | -0.1379813076941497 |
| O50 | 1.0000 | 0.72459 | 0.77541 | 0.15231 | Biso | 1.000 | O | -0.5642717423774206 |
| C145 | 1.0000 | 0.75000 | 0.75000 | 0.13255 | Biso | 1.000 | C | 0.6168858662624891 |
| C146 | 1.0000 | 0.75000 | 0.75000 | 0.08329 | Biso | 1.000 | C | -0.0339681943383726 |
| C147 | 1.0000 | 0.71804 | 0.78196 | 0.06988 | Biso | 1.000 | C | -0.1194810032453188 |
| C148 | 1.0000 | 0.70945 | 0.79055 | 0.02483 | Biso | 1.000 | C | -0.1194810032453188 |
| C149 | 1.0000 | 0.73406 | 0.76594 | 0.99300 | Biso | 1.000 | C | 0.0889764207647183 |
| O51 | 1.0000 | 0.72459 | 0.22459 | 0.84769 | Biso | 1.000 | O | -0.5642717423774206 |
| C150 | 1.0000 | 0.75000 | 0.25000 | 0.86745 | Biso | 1.000 | C | 0.6168858662624891 |
| C151 | 1.0000 | 0.75000 | 0.25000 | 0.91671 | Biso | 1.000 | C | -0.0339681943383726 |
| C152 | 1.0000 | 0.72703 | 0.22703 | 0.94669 | Biso | 1.000 | C | -0.1379813076941497 |
| O52 | 1.0000 | 0.27541 | 0.22459 | 0.15231 | Biso | 1.000 | O | -0.5642717423774206 |
| C153 | 1.0000 | 0.25000 | 0.25000 | 0.13255 | Biso | 1.000 | C | 0.6168858662624891 |
| C154 | 1.0000 | 0.25000 | 0.25000 | 0.08329 | Biso | 1.000 | C | -0.0339681943383726 |
| C155 | 1.0000 | 0.28196 | 0.21804 | 0.06988 | Biso | 1.000 | C | -0.1194810032453188 |
| C156 | 1.0000 | 0.29055 | 0.20945 | 0.02483 | Biso | 1.000 | C | -0.1194810032453188 |
| C157 | 1.0000 | 0.26594 | 0.23406 | 0.99300 | Biso | 1.000 | C | 0.0889764207647183 |
| O53 | 1.0000 | 0.27541 | 0.15231 | 0.22459 | Biso | 1.000 | O | -0.5642717423774206 |
| C158 | 1.0000 | 0.25000 | 0.13255 | 0.25000 | Biso | 1.000 | C | 0.6168858662624891 |
| C159 | 1.0000 | 0.25000 | 0.08329 | 0.25000 | Biso | 1.000 | C | -0.0339681943383726 |
| C160 | 1.0000 | 0.27297 | 0.05331 | 0.22703 | Biso | 1.000 | C | -0.1379813076941497 |
| O54 | 1.0000 | 0.27541 | 0.84769 | 0.77541 | Biso | 1.000 | O | -0.5642717423774206 |
| C161 | 1.0000 | 0.25000 | 0.86745 | 0.75000 | Biso | 1.000 | C | 0.6168858662624891 |
| C162 | 1.0000 | 0.25000 | 0.91671 | 0.75000 | Biso | 1.000 | C | -0.0339681943383726 |
| C163 | 1.0000 | 0.28196 | 0.93012 | 0.78196 | Biso | 1.000 | C | -0.1194810032453188 |
| C164 | 1.0000 | 0.29055 | 0.97517 | 0.79055 | Biso | 1.000 | C | -0.1194810032453188 |
| C165 | 1.0000 | 0.26594 | 0.00700 | 0.76594 | Biso | 1.000 | C | 0.0889764207647183 |
| O55 | 1.0000 | 0.72459 | 0.15231 | 0.77541 | Biso | 1.000 | O | -0.5642717423774206 |
| C166 | 1.0000 | 0.75000 | 0.13255 | 0.75000 | Biso | 1.000 | C | 0.6168858662624891 |
| C167 | 1.0000 | 0.75000 | 0.08329 | 0.75000 | Biso | 1.000 | C | -0.0339681943383726 |
| C168 | 1.0000 | 0.72703 | 0.05331 | 0.77297 | Biso | 1.000 | C | -0.1379813076941497 |
| O56 | 1.0000 | 0.72459 | 0.84769 | 0.22459 | Biso | 1.000 | O | -0.5642717423774206 |
| C169 | 1.0000 | 0.75000 | 0.86745 | 0.25000 | Biso | 1.000 | C | 0.6168858662624891 |
| C170 | 1.0000 | 0.75000 | 0.91671 | 0.25000 | Biso | 1.000 | C | -0.0339681943383726 |
| C171 | 1.0000 | 0.71804 | 0.93012 | 0.21804 | Biso | 1.000 | C | -0.1194810032453188 |
| C172 | 1.0000 | 0.70945 | 0.97517 | 0.20945 | Biso | 1.000 | C | -0.1194810032453188 |
| C173 | 1.0000 | 0.73406 | 0.00700 | 0.23406 | Biso | 1.000 | C | 0.0889764207647183 |
| O57 | 1.0000 | 0.77541 | 0.65231 | 0.77541 | Biso | 1.000 | O | -0.5642717423774206 |
| C174 | 1.0000 | 0.78196 | 0.56988 | 0.78196 | Biso | 1.000 | C | -0.1194810032453188 |
| C175 | 1.0000 | 0.79055 | 0.52483 | 0.79055 | Biso | 1.000 | C | -0.1194810032453188 |
| C176 | 1.0000 | 0.76594 | 0.49300 | 0.76594 | Biso | 1.000 | C | 0.0889764207647183 |
| O58 | 1.0000 | 0.22459 | 0.34769 | 0.77541 | Biso | 1.000 | O | -0.5642717423774206 |
| C177 | 1.0000 | 0.22703 | 0.44669 | 0.77297 | Biso | 1.000 | C | -0.1379813076941497 |
| O59 | 1.0000 | 0.22459 | 0.65231 | 0.22459 | Biso | 1.000 | O | -0.5642717423774206 |
| C178 | 1.0000 | 0.21804 | 0.56988 | 0.21804 | Biso | 1.000 | C | -0.1194810032453188 |
| C179 | 1.0000 | 0.20945 | 0.52483 | 0.20945 | Biso | 1.000 | C | -0.1194810032453188 |
| C180 | 1.0000 | 0.23406 | 0.49300 | 0.23406 | Biso | 1.000 | C | 0.0889764207647183 |
| O60 | 1.0000 | 0.77541 | 0.34769 | 0.22459 | Biso | 1.000 | O | -0.5642717423774206 |
| C181 | 1.0000 | 0.77297 | 0.44669 | 0.22703 | Biso | 1.000 | C | -0.1379813076941497 |

| | | | | | | | | |
|---|---|---|---|---|---|---|---|---|
| O61 | 1.0000 | 0.77541 | 0.27541 | 0.15231 | Biso | 1.000 | O | -0.5642717423774206 |
| O62 | 1.0000 | 0.77541 | 0.72459 | 0.84769 | Biso | 1.000 | O | -0.5642717423774206 |
| C182 | 1.0000 | 0.78196 | 0.71804 | 0.93012 | Biso | 1.000 | C | -0.1194810032453188 |
| C183 | 1.0000 | 0.79055 | 0.70945 | 0.97517 | Biso | 1.000 | C | -0.1194810032453188 |
| C184 | 1.0000 | 0.76594 | 0.73406 | 0.00700 | Biso | 1.000 | C | 0.0889764207647183 |
| O63 | 1.0000 | 0.22459 | 0.72459 | 0.15231 | Biso | 1.000 | O | -0.5642717423774206 |
| C185 | 1.0000 | 0.22703 | 0.72703 | 0.05331 | Biso | 1.000 | C | -0.1379813076941497 |
| O64 | 1.0000 | 0.22459 | 0.27541 | 0.84769 | Biso | 1.000 | O | -0.5642717423774206 |
| C186 | 1.0000 | 0.21804 | 0.28196 | 0.93012 | Biso | 1.000 | C | -0.1194810032453188 |
| C187 | 1.0000 | 0.20945 | 0.29055 | 0.97517 | Biso | 1.000 | C | -0.1194810032453188 |
| C188 | 1.0000 | 0.23406 | 0.26594 | 0.00700 | Biso | 1.000 | C | 0.0889764207647183 |
| O65 | 1.0000 | 0.15231 | 0.27541 | 0.77541 | Biso | 1.000 | O | -0.5642717423774206 |
| C189 | 1.0000 | 0.13255 | 0.25000 | 0.75000 | Biso | 1.000 | C | 0.6168858662624891 |
| C190 | 1.0000 | 0.08329 | 0.25000 | 0.75000 | Biso | 1.000 | C | -0.0339681943383726 |
| C191 | 1.0000 | 0.06988 | 0.28196 | 0.78196 | Biso | 1.000 | C | -0.1194810032453188 |
| C192 | 1.0000 | 0.02483 | 0.29055 | 0.79055 | Biso | 1.000 | C | -0.1194810032453188 |
| C193 | 1.0000 | 0.99300 | 0.26594 | 0.76594 | Biso | 1.000 | C | 0.0889764207647183 |
| O66 | 1.0000 | 0.84769 | 0.27541 | 0.22459 | Biso | 1.000 | O | -0.5642717423774206 |
| C194 | 1.0000 | 0.86745 | 0.25000 | 0.25000 | Biso | 1.000 | C | 0.6168858662624891 |
| C195 | 1.0000 | 0.91671 | 0.25000 | 0.25000 | Biso | 1.000 | C | -0.0339681943383726 |
| C196 | 1.0000 | 0.94669 | 0.27297 | 0.22703 | Biso | 1.000 | C | -0.1379813076941497 |
| O67 | 1.0000 | 0.15231 | 0.72459 | 0.22459 | Biso | 1.000 | O | -0.5642717423774206 |
| C197 | 1.0000 | 0.13255 | 0.75000 | 0.25000 | Biso | 1.000 | C | 0.6168858662624891 |
| C198 | 1.0000 | 0.08329 | 0.75000 | 0.25000 | Biso | 1.000 | C | -0.0339681943383726 |
| C199 | 1.0000 | 0.06988 | 0.71804 | 0.21804 | Biso | 1.000 | C | -0.1194810032453188 |
| C200 | 1.0000 | 0.02483 | 0.70945 | 0.20945 | Biso | 1.000 | C | -0.1194810032453188 |
| C201 | 1.0000 | 0.99300 | 0.73406 | 0.23406 | Biso | 1.000 | C | 0.0889764207647183 |
| O68 | 1.0000 | 0.84769 | 0.72459 | 0.77541 | Biso | 1.000 | O | -0.5642717423774206 |
| C202 | 1.0000 | 0.86745 | 0.75000 | 0.75000 | Biso | 1.000 | C | 0.6168858662624891 |
| C203 | 1.0000 | 0.91671 | 0.75000 | 0.75000 | Biso | 1.000 | C | -0.0339681943383726 |
| C204 | 1.0000 | 0.94669 | 0.72703 | 0.77297 | Biso | 1.000 | C | -0.1379813076941497 |
| O69 | 1.0000 | 0.15231 | 0.27541 | 0.22459 | Biso | 1.000 | O | -0.5642717423774206 |
| C205 | 1.0000 | 0.13255 | 0.25000 | 0.25000 | Biso | 1.000 | C | 0.6168858662624891 |
| C206 | 1.0000 | 0.08329 | 0.25000 | 0.25000 | Biso | 1.000 | C | -0.0339681943383726 |
| C207 | 1.0000 | 0.06988 | 0.28196 | 0.21804 | Biso | 1.000 | C | -0.1194810032453188 |
| C208 | 1.0000 | 0.02483 | 0.29055 | 0.20945 | Biso | 1.000 | C | -0.1194810032453188 |
| C209 | 1.0000 | 0.99300 | 0.26594 | 0.23406 | Biso | 1.000 | C | 0.0889764207647183 |
| O70 | 1.0000 | 0.84769 | 0.72459 | 0.22459 | Biso | 1.000 | O | -0.5642717423774206 |
| C210 | 1.0000 | 0.86745 | 0.75000 | 0.25000 | Biso | 1.000 | C | 0.6168858662624891 |
| C211 | 1.0000 | 0.91671 | 0.75000 | 0.25000 | Biso | 1.000 | C | -0.0339681943383726 |
| C212 | 1.0000 | 0.94669 | 0.72703 | 0.22703 | Biso | 1.000 | C | -0.1379813076941497 |
| O71 | 1.0000 | 0.15231 | 0.72459 | 0.77541 | Biso | 1.000 | O | -0.5642717423774206 |
| C213 | 1.0000 | 0.13255 | 0.75000 | 0.75000 | Biso | 1.000 | C | 0.6168858662624891 |
| C214 | 1.0000 | 0.08329 | 0.75000 | 0.75000 | Biso | 1.000 | C | -0.0339681943383726 |
| C215 | 1.0000 | 0.06988 | 0.71804 | 0.78196 | Biso | 1.000 | C | -0.1194810032453188 |
| C216 | 1.0000 | 0.02483 | 0.70945 | 0.79055 | Biso | 1.000 | C | -0.1194810032453188 |
| C217 | 1.0000 | 0.99300 | 0.73406 | 0.76594 | Biso | 1.000 | C | 0.0889764207647183 |
| O72 | 1.0000 | 0.84769 | 0.27541 | 0.77541 | Biso | 1.000 | O | -0.5642717423774206 |
| C218 | 1.0000 | 0.86745 | 0.25000 | 0.75000 | Biso | 1.000 | C | 0.6168858662624891 |
| C219 | 1.0000 | 0.91671 | 0.25000 | 0.75000 | Biso | 1.000 | C | -0.0339681943383726 |
| C220 | 1.0000 | 0.94669 | 0.27297 | 0.77297 | Biso | 1.000 | C | -0.1379813076941497 |
| O73 | 1.0000 | 0.77541 | 0.27541 | 0.84769 | Biso | 1.000 | O | -0.5642717423774206 |
| C221 | 1.0000 | 0.78196 | 0.28196 | 0.93012 | Biso | 1.000 | C | -0.1194810032453188 |
| C222 | 1.0000 | 0.79055 | 0.29055 | 0.97517 | Biso | 1.000 | C | -0.1194810032453188 |
| C223 | 1.0000 | 0.76594 | 0.26594 | 0.00700 | Biso | 1.000 | C | 0.0889764207647183 |
| O74 | 1.0000 | 0.22459 | 0.27541 | 0.15231 | Biso | 1.000 | O | -0.5642717423774206 |
| C224 | 1.0000 | 0.22703 | 0.27297 | 0.05331 | Biso | 1.000 | C | -0.1379813076941497 |
| O75 | 1.0000 | 0.22459 | 0.72459 | 0.84769 | Biso | 1.000 | O | -0.5642717423774206 |

| | | | | | | | | |
|---|---|---|---|---|---|---|---|---|
| C225 | 1.0000 | 0.21804 | 0.71804 | 0.93012 | Biso | 1.000 | C | -0.1194810032453188 |
| C226 | 1.0000 | 0.20945 | 0.70945 | 0.97517 | Biso | 1.000 | C | -0.1194810032453188 |
| C227 | 1.0000 | 0.23406 | 0.73406 | 0.00700 | Biso | 1.000 | C | 0.0889764207647183 |
| O76 | 1.0000 | 0.77541 | 0.72459 | 0.15231 | Biso | 1.000 | O | -0.5642717423774206 |
| C228 | 1.0000 | 0.77297 | 0.72703 | 0.05331 | Biso | 1.000 | C | -0.1379813076941497 |
| O77 | 1.0000 | 0.77541 | 0.65231 | 0.22459 | Biso | 1.000 | O | -0.5642717423774206 |
| C229 | 1.0000 | 0.78196 | 0.56988 | 0.21804 | Biso | 1.000 | C | -0.1194810032453188 |
| C230 | 1.0000 | 0.79055 | 0.52483 | 0.20945 | Biso | 1.000 | C | -0.1194810032453188 |
| C231 | 1.0000 | 0.76594 | 0.49300 | 0.23406 | Biso | 1.000 | C | 0.0889764207647183 |
| O78 | 1.0000 | 0.77541 | 0.34769 | 0.77541 | Biso | 1.000 | O | -0.5642717423774206 |
| C232 | 1.0000 | 0.77297 | 0.44669 | 0.77297 | Biso | 1.000 | C | -0.1379813076941497 |
| O79 | 1.0000 | 0.22459 | 0.65231 | 0.77541 | Biso | 1.000 | O | -0.5642717423774206 |
| C233 | 1.0000 | 0.21804 | 0.56988 | 0.78196 | Biso | 1.000 | C | -0.1194810032453188 |
| C234 | 1.0000 | 0.20945 | 0.52483 | 0.79055 | Biso | 1.000 | C | -0.1194810032453188 |
| C235 | 1.0000 | 0.23406 | 0.49300 | 0.76594 | Biso | 1.000 | C | 0.0889764207647183 |
| O80 | 1.0000 | 0.22459 | 0.34769 | 0.22459 | Biso | 1.000 | O | -0.5642717423774206 |
| C236 | 1.0000 | 0.22703 | 0.44669 | 0.22703 | Biso | 1.000 | C | -0.1379813076941497 |
| O81 | 1.0000 | 0.77541 | 0.15231 | 0.27541 | Biso | 1.000 | O | -0.5642717423774206 |
| C237 | 1.0000 | 0.78196 | 0.06988 | 0.28196 | Biso | 1.000 | C | -0.1194810032453188 |
| C238 | 1.0000 | 0.79055 | 0.02483 | 0.29055 | Biso | 1.000 | C | -0.1194810032453188 |
| C239 | 1.0000 | 0.76594 | 0.99300 | 0.26594 | Biso | 1.000 | C | 0.0889764207647183 |
| O82 | 1.0000 | 0.22459 | 0.84769 | 0.27541 | Biso | 1.000 | O | -0.5642717423774206 |
| C240 | 1.0000 | 0.22703 | 0.94669 | 0.27297 | Biso | 1.000 | C | -0.1379813076941497 |
| O83 | 1.0000 | 0.22459 | 0.15231 | 0.72459 | Biso | 1.000 | O | -0.5642717423774206 |
| C241 | 1.0000 | 0.21804 | 0.06988 | 0.71804 | Biso | 1.000 | C | -0.1194810032453188 |
| C242 | 1.0000 | 0.20945 | 0.02483 | 0.70945 | Biso | 1.000 | C | -0.1194810032453188 |
| C243 | 1.0000 | 0.23406 | 0.99300 | 0.73406 | Biso | 1.000 | C | 0.0889764207647183 |
| O84 | 1.0000 | 0.77541 | 0.84769 | 0.72459 | Biso | 1.000 | O | -0.5642717423774206 |
| C244 | 1.0000 | 0.77297 | 0.94669 | 0.72703 | Biso | 1.000 | C | -0.1379813076941497 |
| O85 | 1.0000 | 0.77541 | 0.77541 | 0.65231 | Biso | 1.000 | O | -0.5642717423774206 |
| C245 | 1.0000 | 0.77297 | 0.77297 | 0.55331 | Biso | 1.000 | C | -0.1379813076941497 |
| O86 | 1.0000 | 0.77541 | 0.22459 | 0.34769 | Biso | 1.000 | O | -0.5642717423774206 |
| C246 | 1.0000 | 0.79055 | 0.20945 | 0.47517 | Biso | 1.000 | C | -0.1194810032453188 |
| C247 | 1.0000 | 0.76594 | 0.23406 | 0.50700 | Biso | 1.000 | C | 0.0889764207647183 |
| O87 | 1.0000 | 0.22459 | 0.22459 | 0.65231 | Biso | 1.000 | O | -0.5642717423774206 |
| C248 | 1.0000 | 0.22703 | 0.22703 | 0.55331 | Biso | 1.000 | C | -0.1379813076941497 |
| O88 | 1.0000 | 0.22459 | 0.77541 | 0.34769 | Biso | 1.000 | O | -0.5642717423774206 |
| C249 | 1.0000 | 0.21804 | 0.78196 | 0.43012 | Biso | 1.000 | C | -0.1194810032453188 |
| C250 | 1.0000 | 0.20945 | 0.79055 | 0.47517 | Biso | 1.000 | C | -0.1194810032453188 |
| C251 | 1.0000 | 0.23406 | 0.76594 | 0.50700 | Biso | 1.000 | C | 0.0889764207647183 |
| O89 | 1.0000 | 0.15231 | 0.77541 | 0.27541 | Biso | 1.000 | O | -0.5642717423774206 |
| C252 | 1.0000 | 0.05331 | 0.77297 | 0.27297 | Biso | 1.000 | C | -0.1379813076941497 |
| O90 | 1.0000 | 0.84769 | 0.77541 | 0.72459 | Biso | 1.000 | O | -0.5642717423774206 |
| C253 | 1.0000 | 0.93012 | 0.78196 | 0.71804 | Biso | 1.000 | C | -0.1194810032453188 |
| C254 | 1.0000 | 0.97517 | 0.79055 | 0.70945 | Biso | 1.000 | C | -0.1194810032453188 |
| C255 | 1.0000 | 0.00700 | 0.76594 | 0.73406 | Biso | 1.000 | C | 0.0889764207647183 |
| O91 | 1.0000 | 0.15231 | 0.22459 | 0.72459 | Biso | 1.000 | O | -0.5642717423774206 |
| C256 | 1.0000 | 0.05331 | 0.22703 | 0.72703 | Biso | 1.000 | C | -0.1379813076941497 |
| O92 | 1.0000 | 0.84769 | 0.22459 | 0.27541 | Biso | 1.000 | O | -0.5642717423774206 |
| C257 | 1.0000 | 0.93012 | 0.21804 | 0.28196 | Biso | 1.000 | C | -0.1194810032453188 |
| C258 | 1.0000 | 0.97517 | 0.20945 | 0.29055 | Biso | 1.000 | C | -0.1194810032453188 |
| C259 | 1.0000 | 0.00700 | 0.23406 | 0.26594 | Biso | 1.000 | C | 0.0889764207647183 |
| O93 | 1.0000 | 0.15231 | 0.77541 | 0.72459 | Biso | 1.000 | O | -0.5642717423774206 |
| C260 | 1.0000 | 0.05331 | 0.77297 | 0.72703 | Biso | 1.000 | C | -0.1379813076941497 |
| O94 | 1.0000 | 0.84769 | 0.22459 | 0.72459 | Biso | 1.000 | O | -0.5642717423774206 |
| C261 | 1.0000 | 0.93012 | 0.21804 | 0.71804 | Biso | 1.000 | C | -0.1194810032453188 |
| C262 | 1.0000 | 0.97517 | 0.20945 | 0.70945 | Biso | 1.000 | C | -0.1194810032453188 |
| C263 | 1.0000 | 0.00700 | 0.23406 | 0.73406 | Biso | 1.000 | C | 0.0889764207647183 |

| | | | | | | | | |
|---|---|---|---|---|---|---|---|---|
| O95 | 1.0000 | 0.15231 | 0.22459 | 0.27541 | Biso | 1.000 | O | -0.5642717423774206 |
| C264 | 1.0000 | 0.05331 | 0.22703 | 0.27297 | Biso | 1.000 | C | -0.1379813076941497 |
| O96 | 1.0000 | 0.84769 | 0.77541 | 0.27541 | Biso | 1.000 | O | -0.5642717423774206 |
| C265 | 1.0000 | 0.93012 | 0.78196 | 0.28196 | Biso | 1.000 | C | -0.1194810032453188 |
| C266 | 1.0000 | 0.97517 | 0.79055 | 0.29055 | Biso | 1.000 | C | -0.1194810032453188 |
| C267 | 1.0000 | 0.00700 | 0.76594 | 0.26594 | Biso | 1.000 | C | 0.0889764207647183 |
| O97 | 1.0000 | 0.77541 | 0.77541 | 0.34769 | Biso | 1.000 | O | -0.5642717423774206 |
| C268 | 1.0000 | 0.78196 | 0.78196 | 0.43012 | Biso | 1.000 | C | -0.1194810032453188 |
| C269 | 1.0000 | 0.79055 | 0.79055 | 0.47517 | Biso | 1.000 | C | -0.1194810032453188 |
| C270 | 1.0000 | 0.76594 | 0.76594 | 0.50700 | Biso | 1.000 | C | 0.0889764207647183 |
| O98 | 1.0000 | 0.22459 | 0.77541 | 0.65231 | Biso | 1.000 | O | -0.5642717423774206 |
| C271 | 1.0000 | 0.22703 | 0.77297 | 0.55331 | Biso | 1.000 | C | -0.1379813076941497 |
| O99 | 1.0000 | 0.22459 | 0.22459 | 0.34769 | Biso | 1.000 | O | -0.5642717423774206 |
| C272 | 1.0000 | 0.21804 | 0.21804 | 0.43012 | Biso | 1.000 | C | -0.1194810032453188 |
| C273 | 1.0000 | 0.20945 | 0.20945 | 0.47517 | Biso | 1.000 | C | -0.1194810032453188 |
| C274 | 1.0000 | 0.23406 | 0.23406 | 0.50700 | Biso | 1.000 | C | 0.0889764207647183 |
| O100 | 1.0000 | 0.77541 | 0.22459 | 0.65231 | Biso | 1.000 | O | -0.5642717423774206 |
| C275 | 1.0000 | 0.77297 | 0.22703 | 0.55331 | Biso | 1.000 | C | -0.1379813076941497 |
| O101 | 1.0000 | 0.77541 | 0.15231 | 0.72459 | Biso | 1.000 | O | -0.5642717423774206 |
| C276 | 1.0000 | 0.78196 | 0.06988 | 0.71804 | Biso | 1.000 | C | -0.1194810032453188 |
| C277 | 1.0000 | 0.79055 | 0.02483 | 0.70945 | Biso | 1.000 | C | -0.1194810032453188 |
| C278 | 1.0000 | 0.76594 | 0.99300 | 0.73406 | Biso | 1.000 | C | 0.0889764207647183 |
| O102 | 1.0000 | 0.77541 | 0.84769 | 0.27541 | Biso | 1.000 | O | -0.5642717423774206 |
| C279 | 1.0000 | 0.77297 | 0.94669 | 0.27297 | Biso | 1.000 | C | -0.1379813076941497 |
| O103 | 1.0000 | 0.22459 | 0.15231 | 0.27541 | Biso | 1.000 | O | -0.5642717423774206 |
| C280 | 1.0000 | 0.21804 | 0.06988 | 0.28196 | Biso | 1.000 | C | -0.1194810032453188 |
| C281 | 1.0000 | 0.20945 | 0.02483 | 0.29055 | Biso | 1.000 | C | -0.1194810032453188 |
| C282 | 1.0000 | 0.23406 | 0.99300 | 0.26594 | Biso | 1.000 | C | 0.0889764207647183 |
| O104 | 1.0000 | 0.22459 | 0.84769 | 0.72459 | Biso | 1.000 | O | -0.5642717423774206 |
| C283 | 1.0000 | 0.22703 | 0.94669 | 0.72703 | Biso | 1.000 | C | -0.1379813076941497 |
| H1 | 1.0000 | 0.40840 | 0.79816 | 0.79816 | Biso | 1.000 | H | 0.1181960080647832 |
| H2 | 1.0000 | 0.48330 | 0.81212 | 0.81212 | Biso | 1.000 | H | 0.1181960080647832 |
| H3 | 1.0000 | 0.56269 | 0.79428 | 0.79428 | Biso | 1.000 | H | 0.1180356050178862 |
| H4 | 1.0000 | 0.59160 | 0.70184 | 0.70184 | Biso | 1.000 | H | 0.1181960080647832 |
| H5 | 1.0000 | 0.51670 | 0.68788 | 0.68788 | Biso | 1.000 | H | 0.1181960080647832 |
| H6 | 1.0000 | 0.43699 | 0.70579 | 0.70579 | Biso | 1.000 | H | 0.1180356050178862 |
| H7 | 1.0000 | 0.43731 | 0.70572 | 0.29428 | Biso | 1.000 | H | 0.1180356050178862 |
| H8 | 1.0000 | 0.40840 | 0.79816 | 0.20184 | Biso | 1.000 | H | 0.1181960080647832 |
| H9 | 1.0000 | 0.48330 | 0.81212 | 0.18788 | Biso | 1.000 | H | 0.1181960080647832 |
| H10 | 1.0000 | 0.56269 | 0.79428 | 0.20572 | Biso | 1.000 | H | 0.1180356050178862 |
| H11 | 1.0000 | 0.59160 | 0.70184 | 0.29816 | Biso | 1.000 | H | 0.1181960080647832 |
| H12 | 1.0000 | 0.51670 | 0.68788 | 0.31212 | Biso | 1.000 | H | 0.1181960080647832 |
| H13 | 1.0000 | 0.90840 | 0.79816 | 0.29816 | Biso | 1.000 | H | 0.1181960080647832 |
| H14 | 1.0000 | 0.93699 | 0.70579 | 0.20579 | Biso | 1.000 | H | 0.1180356050178862 |
| H15 | 1.0000 | 0.90840 | 0.79816 | 0.70184 | Biso | 1.000 | H | 0.1181960080647832 |
| H16 | 1.0000 | 0.93731 | 0.70572 | 0.79428 | Biso | 1.000 | H | 0.1180356050178862 |
| H17 | 1.0000 | 0.43699 | 0.29421 | 0.29421 | Biso | 1.000 | H | 0.1180356050178862 |
| H18 | 1.0000 | 0.51670 | 0.31212 | 0.31212 | Biso | 1.000 | H | 0.1181960080647832 |
| H19 | 1.0000 | 0.59160 | 0.29816 | 0.29816 | Biso | 1.000 | H | 0.1181960080647832 |
| H20 | 1.0000 | 0.56269 | 0.20572 | 0.20572 | Biso | 1.000 | H | 0.1180356050178862 |
| H21 | 1.0000 | 0.48330 | 0.18788 | 0.18788 | Biso | 1.000 | H | 0.1181960080647832 |
| H22 | 1.0000 | 0.40840 | 0.20184 | 0.20184 | Biso | 1.000 | H | 0.1181960080647832 |
| H23 | 1.0000 | 0.51670 | 0.31212 | 0.68788 | Biso | 1.000 | H | 0.1181960080647832 |
| H24 | 1.0000 | 0.59160 | 0.29816 | 0.70184 | Biso | 1.000 | H | 0.1181960080647832 |
| H25 | 1.0000 | 0.56269 | 0.20572 | 0.79428 | Biso | 1.000 | H | 0.1180356050178862 |
| H26 | 1.0000 | 0.48330 | 0.18788 | 0.81212 | Biso | 1.000 | H | 0.1181960080647832 |
| H27 | 1.0000 | 0.40840 | 0.20184 | 0.79816 | Biso | 1.000 | H | 0.1181960080647832 |
| H28 | 1.0000 | 0.43731 | 0.29428 | 0.70572 | Biso | 1.000 | H | 0.1180356050178862 |

| | | | | | | | | |
|---|---|---|---|---|---|---|---|---|
| H29 | 1.0000 | 0.20184 | 0.59160 | 0.79816 | Biso | 1.000 | H | 0.1181960080647832 |
| H30 | 1.0000 | 0.18788 | 0.51670 | 0.81212 | Biso | 1.000 | H | 0.1181960080647832 |
| H31 | 1.0000 | 0.20572 | 0.43731 | 0.79428 | Biso | 1.000 | H | 0.1180356050178862 |
| H32 | 1.0000 | 0.31212 | 0.48330 | 0.68788 | Biso | 1.000 | H | 0.1181960080647832 |
| H33 | 1.0000 | 0.29816 | 0.40840 | 0.29816 | Biso | 1.000 | H | 0.1181960080647832 |
| H34 | 1.0000 | 0.31212 | 0.48330 | 0.31212 | Biso | 1.000 | H | 0.1181960080647832 |
| H35 | 1.0000 | 0.29428 | 0.56269 | 0.29428 | Biso | 1.000 | H | 0.1180356050178862 |
| H36 | 1.0000 | 0.20184 | 0.59160 | 0.20184 | Biso | 1.000 | H | 0.1181960080647832 |
| H37 | 1.0000 | 0.18788 | 0.51670 | 0.18788 | Biso | 1.000 | H | 0.1181960080647832 |
| H38 | 1.0000 | 0.20572 | 0.43731 | 0.20572 | Biso | 1.000 | H | 0.1180356050178862 |
| H39 | 1.0000 | 0.68788 | 0.48330 | 0.68788 | Biso | 1.000 | H | 0.1181960080647832 |
| H40 | 1.0000 | 0.70184 | 0.40840 | 0.70184 | Biso | 1.000 | H | 0.1181960080647832 |
| H41 | 1.0000 | 0.70572 | 0.56269 | 0.70572 | Biso | 1.000 | H | 0.1180356050178862 |
| H42 | 1.0000 | 0.79816 | 0.59160 | 0.79816 | Biso | 1.000 | H | 0.1181960080647832 |
| H43 | 1.0000 | 0.81212 | 0.51670 | 0.81212 | Biso | 1.000 | H | 0.1181960080647832 |
| H44 | 1.0000 | 0.79428 | 0.43731 | 0.79428 | Biso | 1.000 | H | 0.1180356050178862 |
| H45 | 1.0000 | 0.70184 | 0.40840 | 0.29816 | Biso | 1.000 | H | 0.1181960080647832 |
| H46 | 1.0000 | 0.68788 | 0.48330 | 0.31212 | Biso | 1.000 | H | 0.1181960080647832 |
| H47 | 1.0000 | 0.79428 | 0.43731 | 0.20572 | Biso | 1.000 | H | 0.1180356050178862 |
| H48 | 1.0000 | 0.81212 | 0.51670 | 0.18788 | Biso | 1.000 | H | 0.1181960080647832 |
| H49 | 1.0000 | 0.79816 | 0.59160 | 0.20184 | Biso | 1.000 | H | 0.1181960080647832 |
| H50 | 1.0000 | 0.70579 | 0.56301 | 0.29421 | Biso | 1.000 | H | 0.1180356050178862 |
| H51 | 1.0000 | 0.93731 | 0.29428 | 0.20572 | Biso | 1.000 | H | 0.1180356050178862 |
| H52 | 1.0000 | 0.90840 | 0.20184 | 0.29816 | Biso | 1.000 | H | 0.1181960080647832 |
| H53 | 1.0000 | 0.93699 | 0.29421 | 0.79421 | Biso | 1.000 | H | 0.1180356050178862 |
| H54 | 1.0000 | 0.90840 | 0.20184 | 0.70184 | Biso | 1.000 | H | 0.1181960080647832 |
| H55 | 1.0000 | 0.79816 | 0.09160 | 0.29816 | Biso | 1.000 | H | 0.1181960080647832 |
| H56 | 1.0000 | 0.70572 | 0.06269 | 0.20572 | Biso | 1.000 | H | 0.1180356050178862 |
| H57 | 1.0000 | 0.79816 | 0.09160 | 0.70184 | Biso | 1.000 | H | 0.1181960080647832 |
| H58 | 1.0000 | 0.70579 | 0.06301 | 0.79421 | Biso | 1.000 | H | 0.1180356050178862 |
| H59 | 1.0000 | 0.29421 | 0.06301 | 0.20579 | Biso | 1.000 | H | 0.1180356050178862 |
| H60 | 1.0000 | 0.20184 | 0.09160 | 0.29816 | Biso | 1.000 | H | 0.1181960080647832 |
| H61 | 1.0000 | 0.29428 | 0.06269 | 0.79428 | Biso | 1.000 | H | 0.1180356050178862 |
| H62 | 1.0000 | 0.20184 | 0.09160 | 0.70184 | Biso | 1.000 | H | 0.1181960080647832 |
| H63 | 1.0000 | 0.06269 | 0.20572 | 0.70572 | Biso | 1.000 | H | 0.1180356050178862 |
| H64 | 1.0000 | 0.09160 | 0.29816 | 0.79816 | Biso | 1.000 | H | 0.1181960080647832 |
| H65 | 1.0000 | 0.06269 | 0.20572 | 0.29428 | Biso | 1.000 | H | 0.1180356050178862 |
| H66 | 1.0000 | 0.09160 | 0.29816 | 0.20184 | Biso | 1.000 | H | 0.1181960080647832 |
| H67 | 1.0000 | 0.09160 | 0.70184 | 0.79816 | Biso | 1.000 | H | 0.1181960080647832 |
| H68 | 1.0000 | 0.06269 | 0.79428 | 0.70572 | Biso | 1.000 | H | 0.1180356050178862 |
| H69 | 1.0000 | 0.06269 | 0.79428 | 0.29428 | Biso | 1.000 | H | 0.1180356050178862 |
| H70 | 1.0000 | 0.20572 | 0.93731 | 0.70572 | Biso | 1.000 | H | 0.1180356050178862 |
| H71 | 1.0000 | 0.09160 | 0.70184 | 0.20184 | Biso | 1.000 | H | 0.1181960080647832 |
| H72 | 1.0000 | 0.29816 | 0.90840 | 0.20184 | Biso | 1.000 | H | 0.1181960080647832 |
| H73 | 1.0000 | 0.29816 | 0.90840 | 0.79816 | Biso | 1.000 | H | 0.1181960080647832 |
| H74 | 1.0000 | 0.20572 | 0.93731 | 0.29428 | Biso | 1.000 | H | 0.1180356050178862 |
| H75 | 1.0000 | 0.79428 | 0.93731 | 0.29428 | Biso | 1.000 | H | 0.1180356050178862 |
| H76 | 1.0000 | 0.70184 | 0.90840 | 0.20184 | Biso | 1.000 | H | 0.1181960080647832 |
| H77 | 1.0000 | 0.79428 | 0.93731 | 0.70572 | Biso | 1.000 | H | 0.1180356050178862 |
| H78 | 1.0000 | 0.70184 | 0.90840 | 0.79816 | Biso | 1.000 | H | 0.1181960080647832 |
| H79 | 1.0000 | 0.18788 | 0.81212 | 0.48330 | Biso | 1.000 | H | 0.1181960080647832 |
| H80 | 1.0000 | 0.20184 | 0.79816 | 0.40840 | Biso | 1.000 | H | 0.1181960080647832 |
| H81 | 1.0000 | 0.20572 | 0.79428 | 0.56269 | Biso | 1.000 | H | 0.1180356050178862 |
| H82 | 1.0000 | 0.29816 | 0.70184 | 0.59160 | Biso | 1.000 | H | 0.1181960080647832 |
| H83 | 1.0000 | 0.31212 | 0.68788 | 0.51670 | Biso | 1.000 | H | 0.1181960080647832 |
| H84 | 1.0000 | 0.29428 | 0.70572 | 0.43731 | Biso | 1.000 | H | 0.1180356050178862 |
| H85 | 1.0000 | 0.81212 | 0.81212 | 0.48330 | Biso | 1.000 | H | 0.1181960080647832 |
| H86 | 1.0000 | 0.79816 | 0.79816 | 0.40840 | Biso | 1.000 | H | 0.1181960080647832 |

| | | | | | | | | |
|---|---|---|---|---|---|---|---|---|
| H87 | 1.0000 | 0.70579 | 0.70579 | 0.43699 | Biso | 1.000 | H | 0.1180356050178862 |
| H88 | 1.0000 | 0.79428 | 0.79428 | 0.56269 | Biso | 1.000 | H | 0.1180356050178862 |
| H89 | 1.0000 | 0.70184 | 0.70184 | 0.59160 | Biso | 1.000 | H | 0.1181960080647832 |
| H90 | 1.0000 | 0.68788 | 0.68788 | 0.51670 | Biso | 1.000 | H | 0.1181960080647832 |
| H91 | 1.0000 | 0.29421 | 0.29421 | 0.43699 | Biso | 1.000 | H | 0.1180356050178862 |
| H92 | 1.0000 | 0.31212 | 0.31212 | 0.51670 | Biso | 1.000 | H | 0.1181960080647832 |
| H93 | 1.0000 | 0.29816 | 0.29816 | 0.59160 | Biso | 1.000 | H | 0.1181960080647832 |
| H94 | 1.0000 | 0.20572 | 0.20572 | 0.56269 | Biso | 1.000 | H | 0.1180356050178862 |
| H95 | 1.0000 | 0.18788 | 0.18788 | 0.48330 | Biso | 1.000 | H | 0.1181960080647832 |
| H96 | 1.0000 | 0.20184 | 0.20184 | 0.40840 | Biso | 1.000 | H | 0.1181960080647832 |
| H97 | 1.0000 | 0.68788 | 0.31212 | 0.51670 | Biso | 1.000 | H | 0.1181960080647832 |
| H98 | 1.0000 | 0.70572 | 0.29428 | 0.43731 | Biso | 1.000 | H | 0.1180356050178862 |
| H99 | 1.0000 | 0.70184 | 0.29816 | 0.59160 | Biso | 1.000 | H | 0.1181960080647832 |
| H100 | 1.0000 | 0.79428 | 0.20572 | 0.56269 | Biso | 1.000 | H | 0.1180356050178862 |
| H101 | 1.0000 | 0.81212 | 0.18788 | 0.48330 | Biso | 1.000 | H | 0.1181960080647832 |
| H102 | 1.0000 | 0.79428 | 0.70572 | 0.06269 | Biso | 1.000 | H | 0.1180356050178862 |
| H103 | 1.0000 | 0.70184 | 0.79816 | 0.09160 | Biso | 1.000 | H | 0.1181960080647832 |
| H104 | 1.0000 | 0.20572 | 0.29428 | 0.06269 | Biso | 1.000 | H | 0.1180356050178862 |
| H105 | 1.0000 | 0.29816 | 0.20184 | 0.09160 | Biso | 1.000 | H | 0.1181960080647832 |
| H106 | 1.0000 | 0.29816 | 0.79816 | 0.09160 | Biso | 1.000 | H | 0.1181960080647832 |
| H107 | 1.0000 | 0.20572 | 0.70572 | 0.06269 | Biso | 1.000 | H | 0.1180356050178862 |
| H108 | 1.0000 | 0.79816 | 0.29816 | 0.90840 | Biso | 1.000 | H | 0.1181960080647832 |
| H109 | 1.0000 | 0.70579 | 0.20579 | 0.93699 | Biso | 1.000 | H | 0.1180356050178862 |
| H110 | 1.0000 | 0.70572 | 0.79428 | 0.93731 | Biso | 1.000 | H | 0.1180356050178862 |
| H111 | 1.0000 | 0.79816 | 0.70184 | 0.90840 | Biso | 1.000 | H | 0.1181960080647832 |
| H112 | 1.0000 | 0.29421 | 0.79421 | 0.93699 | Biso | 1.000 | H | 0.1180356050178862 |
| H113 | 1.0000 | 0.20184 | 0.70184 | 0.90840 | Biso | 1.000 | H | 0.1181960080647832 |
| H114 | 1.0000 | 0.20184 | 0.29816 | 0.90840 | Biso | 1.000 | H | 0.1181960080647832 |
| H115 | 1.0000 | 0.29428 | 0.20572 | 0.93731 | Biso | 1.000 | H | 0.1180356050178862 |
| H116 | 1.0000 | 0.01670 | 0.68788 | 0.18788 | Biso | 1.000 | H | 0.1181960080647832 |
| H117 | 1.0000 | 0.01670 | 0.68788 | 0.81212 | Biso | 1.000 | H | 0.1181960080647832 |
| H118 | 1.0000 | 0.01670 | 0.31212 | 0.18788 | Biso | 1.000 | H | 0.1181960080647832 |
| H119 | 1.0000 | 0.01670 | 0.31212 | 0.81212 | Biso | 1.000 | H | 0.1181960080647832 |
| H120 | 1.0000 | 0.18788 | 0.01670 | 0.31212 | Biso | 1.000 | H | 0.1181960080647832 |
| H121 | 1.0000 | 0.18788 | 0.01670 | 0.68788 | Biso | 1.000 | H | 0.1181960080647832 |
| H122 | 1.0000 | 0.81212 | 0.01670 | 0.31212 | Biso | 1.000 | H | 0.1181960080647832 |
| H123 | 1.0000 | 0.81212 | 0.01670 | 0.68788 | Biso | 1.000 | H | 0.1181960080647832 |
| H124 | 1.0000 | 0.98330 | 0.18788 | 0.31212 | Biso | 1.000 | H | 0.1181960080647832 |
| H125 | 1.0000 | 0.98330 | 0.18788 | 0.68788 | Biso | 1.000 | H | 0.1181960080647832 |
| H126 | 1.0000 | 0.98330 | 0.81212 | 0.68788 | Biso | 1.000 | H | 0.1181960080647832 |
| H127 | 1.0000 | 0.98330 | 0.81212 | 0.31212 | Biso | 1.000 | H | 0.1181960080647832 |
| H128 | 1.0000 | 0.68788 | 0.98330 | 0.81212 | Biso | 1.000 | H | 0.1181960080647832 |
| H129 | 1.0000 | 0.68788 | 0.98330 | 0.18788 | Biso | 1.000 | H | 0.1181960080647832 |
| H130 | 1.0000 | 0.31212 | 0.98330 | 0.81212 | Biso | 1.000 | H | 0.1181960080647832 |
| H131 | 1.0000 | 0.31212 | 0.98330 | 0.18788 | Biso | 1.000 | H | 0.1181960080647832 |
| H132 | 1.0000 | 0.68788 | 0.18788 | 0.01670 | Biso | 1.000 | H | 0.1181960080647832 |
| H133 | 1.0000 | 0.31212 | 0.18788 | 0.01670 | Biso | 1.000 | H | 0.1181960080647832 |
| H134 | 1.0000 | 0.68788 | 0.81212 | 0.01670 | Biso | 1.000 | H | 0.1181960080647832 |
| H135 | 1.0000 | 0.31212 | 0.81212 | 0.01670 | Biso | 1.000 | H | 0.1181960080647832 |
| H136 | 1.0000 | 0.81212 | 0.68788 | 0.98330 | Biso | 1.000 | H | 0.1181960080647832 |
| H137 | 1.0000 | 0.18788 | 0.68788 | 0.98330 | Biso | 1.000 | H | 0.1181960080647832 |
| H138 | 1.0000 | 0.18788 | 0.31212 | 0.98330 | Biso | 1.000 | H | 0.1181960080647832 |
| H139 | 1.0000 | 0.81212 | 0.31212 | 0.98330 | Biso | 1.000 | H | 0.1181960080647832 |
| C284 | 1.0000 | 0.27297 | 0.55331 | 0.72703 | Biso | 1.000 | C | -0.1093502611168461 |
| H140 | 1.0000 | 0.29644 | 0.56608 | 0.70356 | Biso | 1.000 | H | 0.1096151416674911 |
| C285 | 1.0000 | 0.28196 | 0.43012 | 0.71804 | Biso | 1.000 | C | -0.1012118711223034 |
| H141 | 1.0000 | 0.30012 | 0.40553 | 0.69988 | Biso | 1.000 | H | 0.1096151416674911 |
| C286 | 1.0000 | 0.78196 | 0.21804 | 0.43012 | Biso | 1.000 | C | -0.1012118711223034 |

| | | | | | | | | |
|---|---|---|---|---|---|---|---|---|
| H142 | 1.0000 | 0.80012 | 0.19988 | 0.40553 | Biso | 1.000 | H | 0.1096151416674911 |
| C287 | 1.0000 | 0.71804 | 0.21804 | 0.06988 | Biso | 1.000 | C | -0.1012118711223034 |
| H143 | 1.0000 | 0.69988 | 0.19988 | 0.09447 | Biso | 1.000 | H | 0.1096151416674911 |
| C288 | 1.0000 | 0.77297 | 0.27297 | 0.05331 | Biso | 1.000 | C | -0.1093502611168461 |
| H144 | 1.0000 | 0.79644 | 0.29644 | 0.06608 | Biso | 1.000 | H | 0.1096151416674911 |
